# Therapeutic targeting of TGF‐β in lung cancer

**DOI:** 10.1111/febs.17234

**Published:** 2024-07-31

**Authors:** Sajjad Aftabi, Amir Barzegar Behrooz, Marco Cordani, Niloufar Rahiman, Mohammadamin Sadeghdoust, Farnaz Aligolighasemabadi, Stephen Pistorius, Seyedeh Hoda Alavizadeh, Nima Taefehshokr, Saeid Ghavami

**Affiliations:** ^1^ Department of Human Anatomy and Cell Science University of Manitoba College of Medicine Winnipeg Canada; ^2^ Paul Albrechtsen Research Institute, CancerCare Manitoba University of Manitoba Winnipeg Canada; ^3^ Department of Physics and Astronomy University of Manitoba Winnipeg Canada; ^4^ Electrophysiology Research Center, Neuroscience Institute Tehran University of Medical Sciences Iran; ^5^ Department of Biochemistry and Molecular Biology, Faculty of Biology Complutense University Madrid Spain; ^6^ Instituto de Investigaciones Sanitarias San Carlos (IdISSC) Madrid Spain; ^7^ Nanotechnology Research Center, Pharmaceutical Technology Institute Mashhad University of Medical Sciences Iran; ^8^ Department of Pharmaceutical Nanotechnology, School of Pharmacy Mashhad University of Medical Sciences Iran; ^9^ Division of BioMedical Sciences, Faculty of Medicine Memorial University of Newfoundland St. John's Canada; ^10^ Apoptosis Research Centre Children's Hospital of Eastern Ontario Research Institute Ottawa Canada; ^11^ Faculty Academy of Silesia, Faculty of Medicine Katowice Poland; ^12^ Children Hospital Research Institute of Manitoba University of Manitoba Winnipeg Canada

**Keywords:** chemoresistance, epithelial–mesenchymal transition, immunotherapy, lung cancer, transforming growth factor‐β

## Abstract

Transforming growth factor‐β (TGF‐β) plays a complex role in lung cancer pathophysiology, initially acting as a tumor suppressor by inhibiting early‐stage tumor growth. However, its role evolves in the advanced stages of the disease, where it contributes to tumor progression not by directly promoting cell proliferation but by enhancing epithelial–mesenchymal transition (EMT) and creating a conducive tumor microenvironment. While EMT is typically associated with enhanced migratory and invasive capabilities rather than proliferation *per se*, TGF‐β's influence on this process facilitates the complex dynamics of tumor metastasis. Additionally, TGF‐β impacts the tumor microenvironment by interacting with immune cells, a process influenced by genetic and epigenetic changes within tumor cells. This interaction highlights its role in immune evasion and chemoresistance, further complicating lung cancer therapy. This review provides a critical overview of recent findings on TGF‐β's involvement in lung cancer, its contribution to chemoresistance, and its modulation of the immune response. Despite the considerable challenges encountered in clinical trials and the development of new treatments targeting the TGF‐β pathway, this review highlights the necessity for continued, in‐depth investigation into the roles of TGF‐β. A deeper comprehension of these roles may lead to novel, targeted therapies for lung cancer. Despite the intricate behavior of TGF‐β signaling in tumors and previous challenges, further research could yield innovative treatment strategies.

## Graphical abstract

Here, we discuss how lung cancer treatment can be transformed by targeting TGF‐β for better outcomes. Inhibiting TGF‐β signaling halts tumor growth, boosts immune response, and enhances treatment efficacy. By tackling TGF‐β's role in epithelial–mesenchymal transition (EMT) and immune evasion, this approach opens new avenues for more effective lung cancer therapies and improved patient outcomes, offering hope for a brighter future in lung cancer treatment and patient care.

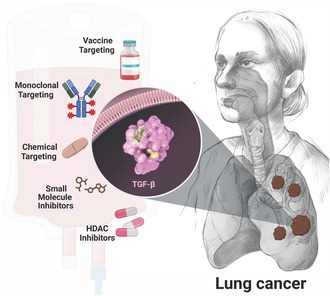

**Abbreviations**
A549: human alveolar basal epithelial cell line
AKT: protein kinase B
ALK5: activin receptor‐like kinase 5
APCs: antigen‐presenting cells
ASCL1: achaete‐scute family BHLH transcription factor 1
ATF‐1: activating transcription factor 1
Bcl‐2, BIM: B‐cell CLL/lymphoma 2, Bcl‐2‐like protein 11
BMP: bone morphogenetic protein
BUB1: budding uninhibited by benzimidazoles 1
CAF: cancer‐associated fibroblast
CD4+: cluster of differentiation 4 positive
CD437: retinoic acid receptor agonist
CD8+: cluster of differentiation 8 positive
CDP138: C2 domain‐containing phosphoprotein
CIITA: class II transactivator
COX‐2: cyclooxygenase‐2
CTL: cytotoxic T lymphocyte
CTLA‐4: cytotoxic T‐lymphocyte‐associated protein 4
CXCL9, CXCL10: C‐X‐C motif chemokine ligand 9, 10
CXCR3: C‐X‐C motif chemokine receptor 3
DC: dendritic cells
DN‐TGFβRII: dominant negative transforming growth factor beta receptor type II
E2F1: E2F transcription factor 1
ECD: extracellular domain
ECM: extracellular matrix
EEF1A2: eukaryotic translation elongation factor 1 alpha 2
EGFR: epidermal growth factor receptor
EGFR‐TKI: epidermal growth factor receptor‐tyrosine kinase inhibitor
EMT: epithelial–mesenchymal transition
ERK: extracellular signal‐regulated kinase
Foxp3+: forkhead box P3 positive
GDF15: growth differentiation factor 15
GM‐CSF: granulocyte‐macrophage colony‐stimulating factor
GRB2: growth factor receptor‐bound protein 2
GS: glycine–serine
GSK3: glycogen synthase kinase 3
H460: human non‐small‐cell lung carcinoma cell line
HDAC: histone deacetylase
HMG‐CoA: 3‐hydroxy‐3‐methylglutaryl‐coenzyme A
Id1: inhibitor of differentiation 1
IDO: indoleamine 2,3‐dioxygenase
IFN‐γ: interferon gamma
IGFII: insulin‐like growth factor II
IL‐2, IL‐21, IL‐4, IL‐6, IL‐10, IL‐12, IL‐17: interleukins 2, 21, 4, 6, 10, 12, 17
ILC‐1: type 1 innate lymphoid cells
IRAK‐M: interleukin receptor‐associated kinase M
IRF8: interferon regulatory factor 8
JNK: c‐Jun N‐terminal kinase
LAP: latency‐associated peptide
LCNEC: large cell neuroendocrine carcinoma
LTBP: latency binding protein
M2: macrophage type 2
Mad: mothers against Dpp protein family
MAPK: mitogen‐activated protein kinase
MDSCs: myeloid‐derived suppress cells

## Introduction

Lung cancer is the most prevalent and lethal type of cancer on a global scale [1]. Certain demographics, such as men, people over the age of 60, African Americans, and those with a family history, are more prone to developing lung cancer. Depending on the origin of the cancer cells, lung cancer can be classified as small‐cell lung cancer (SCLC) and non‐small‐cell lung cancer (NSCLC). The predominant subtypes of lung cancer include adenocarcinoma, squamous cell carcinoma (SCC), and neuroendocrine tumors such as small cell carcinoma (SCLC), large cell neuroendocrine carcinoma (LCNEC), and carcinoid [2, 3].

The transforming growth factor beta (TGF‐β) superfamily consists of multifarious cytokines, including TGFβ isoforms, bone morphogenetic proteins (BMPs), and activins that modulate embryonic development and hormone release [4, 5, 6, 7]. Numerous intricate mechanisms steer the biosynthesis, secretion, and activation of TGF‐β; any deregulation in TGF‐β family signaling has been associated with a plethora of diseases [8]. The lungs, exposed to numerous external stimuli such as pollutants and pathogens, rely on TGF‐β to maintain tissue structure (organogenesis) and normal functioning (homeostasis) and prevent cancer development (tumorigenesis) [9, 10]. TGF‐β can either promote or inhibit the growth of lung tissue cells depending on the context, complicating its role in lung diseases, including cancer [9, 11].

The role of TGF‐β in tumorigenesis is paradoxical. Initially, it preserves the normal structure of lung tissue by preventing excessive cell proliferation and affecting the tumor's surrounding environment to inhibit cancer development [9, 12]. However, if TGF‐β signaling becomes disrupted, it may promote a phenomenon known as epithelial‐to‐mesenchymal transition (EMT), enabling cancer cells to invade other tissues and establish new tumors in distant organs (metastasis) [13, 14]. Additionally, TGF‐β is integral to immune system regulation, influencing immune homeostasis and evasion, thus reshaping the tumor microenvironment [9, 15].

Recognizing the multifaceted role of TGF‐β in tumorigenesis opens avenues for potential therapeutic interventions in lung cancer, particularly through targeted immune therapies. For instance, drugs like galunisertib are designed to correct the faulty TGF‐β signaling contributing to cancer progression [16, 17]. On the immunotherapy front, approaches like M7824 block the effects of TGF‐β that prevent the immune system from attacking cancer cells, thus enhancing the body's ability to fight cancer [18].

This review focuses on the involvement of TGF‐β in regulating the immune system and its significance in the context of lung cancer. This paper provides an overview of the structure, synthesis, maturation, and degradation, along with a summary of current knowledge about the signaling pathways, focusing on its role in lung development, hemostasis, and lung cancer. We review the transition of TGF‐β effects from tumor‐suppressive to pro‐metastatic throughout the progression of lung malignancies. Additionally, we highlight TGF‐β's significant impact on key cellular components of both the innate and adaptive immune systems. Finally, we discuss potential TGFβ‐related therapies for lung cancer. This section reviews recent advances in how lung cancer cells exploit the immunosuppressive properties of TGF‐β to evade detection by the immune system and resist cancer immunotherapy. Understanding these mechanisms more clearly could lead to breakthroughs in lung cancer treatment by targeting these evasion strategies.

## From structural to functional perspectives of TGF‐β

### Structures of TGF‐β isoforms and their receptors

**Fig. 1:**
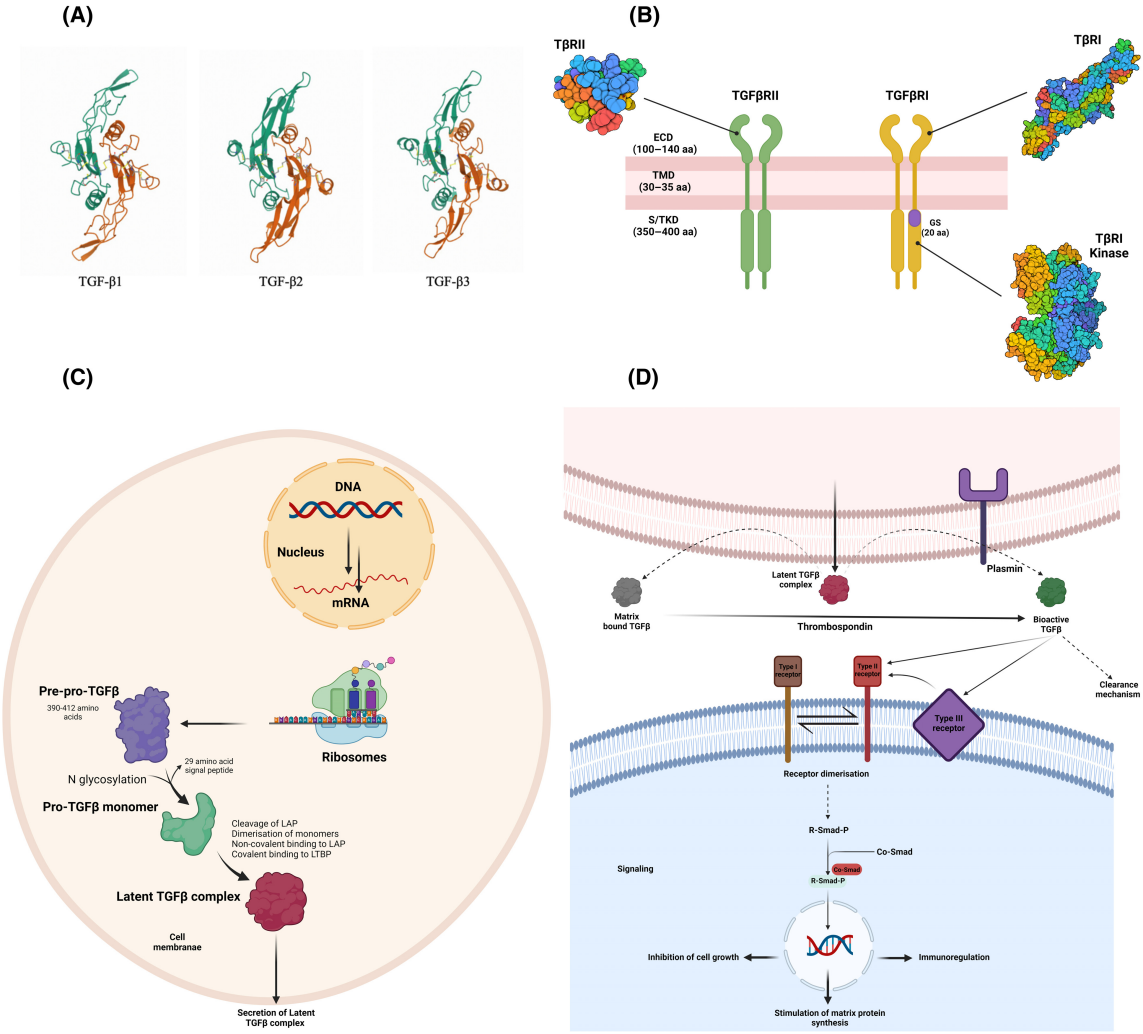
From structural to functional perspectives of TGF‐β. (A) Structures of three TGF‐β isoforms. Structures of three TGF‐β isoforms described as the palm (central a‐helix of one monomer) of one curved left‐hand packs against the heel (concave surface of the extended b‐strands of the other monomer) of the other hand. PDB ID (http://www.wwpdb.org/) used for each structure: TGF‐β1 (1KLC), TGF‐β2 (2TGI), and TGF‐β3 (1TGK) [19]. TGF‐β1 regulates immune responses and fibrosis, crucial in wound healing. TGF‐β2 is vital for embryonic development, affecting heart, lung, and CNS formation. TGF‐β3 plays key roles in palatogenesis, wound healing, and tissue regeneration, with distinct but overlapping functions with other isoforms [20, 21]. (B) Structure of TGFβRI and TGFβRII. The receptors' extracellular domain (ECD) has 100–140 residues, forming a three‐leg toxin fold (F1, F2, and F3). The receptors' transmembrane domain (TMD) comprises 30–35 residues, while cytoplasmic serine–threonine kinase domains (S/TKD) consist of 350–400 residues. The purple region in S/TKD of TGFβRI shows the juxtamembrane glycine–serine‐rich regulatory domain, that is, GS domain, that consists of 20 residues. The TβRI ECD, TβRII ECD, and TβRI kinase structures come from PDB entries 2PJY, 1M9Z, and 1IAS, respectively. PDB ID (http://www.wwpdb.org/) [7]. (C) Latent TGF‐β1 synthesis and secretion [22]. (D) From latent TGF‐β1 release in the extracellular environment to its activation and signaling pathway. Released latent TGF‐β can attach to an extracellular matrix or a cell membrane‐associated mannose‐6‐phosphate/insulin‐like growth factor II receptor, waiting for activation. As shown here, proteolytic enzymes such as plasmin and thrombospondin can catalyze the activation process of latent TGF‐β [22]. IGFII, insulin‐like growth factor II; LAP, latency‐associated peptide containing mannose‐6‐phosphate residues; LTBP, latent TGF‐β binding protein; M6P, mannose 6 phosphate; Mad, mothers against dpp protein family; TGFβ, transforming growth factor beta (Created with BioRender.com).

TGF‐β superfamily proteins possess a dimeric structure held together by an inter‐chain disulfide bond, ensuring stability. TGF‐β in humans exists in three isoforms: TGF‐β1, TGF‐β2, and TGF‐β3 (Fig. 1A). Despite sharing 60–80% sequence homology, these isoforms exhibit slight functional differences. Initially, they are synthesized as inactive precursors known as latent‐TGF‐β, which undergo intracellular modifications before secretion [23].

TGF‐β1 consists of 390 amino acids, whereas TGF‐β2 and TGF‐β3 each have 412 amino acids [7]. They are derived from distinct genes, comprising an N‐terminal peptide, a latency‐associated peptide (LAP) pro‐region, and a C‐terminal active region. Proteolytic cleavage by furin produces two dimers: a 65–75 kDa LAP pro‐region and a 25 kDa active TGF‐β [24]. The mature TGF‐β consists of two 11.5–12.5 kDa polypeptide chains. There are nine cysteine residues in TGF‐β, with eight forming a cysteine knot in monomers and the ninth contributing to dimerization [15]. TGF‐β receptor complex consists of two type I (TGFβRI also known as ALK‐5) and two type II (TGFβRII) receptor dimers that form a hetero‐tetramer upon TGF‐β binding the type II receptor. Both receptors are transmembrane kinases with a cysteine‐abundant extracellular domain, a transmembrane domain, and a high abundance of intracellular serine/threonine domain (Fig. 1B) [25]. The type I receptor has a glycine–serine‐rich (GS) domain phosphorylated by the type II receptor upon the TGF‐β ligand family binding the type II receptor dimer in the extracellular domain [26]. In the absence of TGF‐β, the TGFβRI and TGFβRII exist as monomers on the cell surface rather than homodimers [27]. The TGFβRII is found to function as constitutively active kinases [25]. Upon TGF‐β binding to TGFβRII, conformational changes occur, resulting in dimer formation and phosphorylation of the type I receptors in their GS domain [28]. This triggers TGFβRI activation and a downstream signaling cascade involving SMAD proteins [25]. To fully activate TGFβRI, at least three phosphorylations of serine and threonine residues are required in the GS domain [29]. The specificity of the heteromeric type I and type II TGF‐β receptor complexes is characterized by the ectodomain of the type II receptors that exclusively bind to TGF‐β ligands and the type I receptor kinases that initiate the signaling responses [28].

### Synthesis, maturation, and degradation

TGF‐β1 is generated from a gene that is initially transcribed into mRNA and subsequently translated into a precursor protein consisting of 390–412 amino acids. This precursor protein undergoes a modification known as N‐Glycosylation. After this modification, it is cleaved by enzymes like furin to produce pro‐TGF‐β, which remains biologically inactive until it is needed by the body. In this inactive form, TGF‐β is bound to a protein called latency‐associated peptide (LAP), preventing its activity until specific cellular signals trigger its activation [30]. Key residues in TGF‐β1 LAP, particularly cysteine at positions 223 and 225, are crucial for its inactivation by binding noncovalently to TGF‐β1 [29]. The third cysteine links to the latency binding protein (LTBP) that aids in anchoring latent TGF‐β to the extracellular matrix, thus serving as a TGF‐β reservoir [29]. This matrix‐bound latent TGF‐β can be activated through various conditions and agents, such as extreme pH, high temperatures, and proteolytic enzymes [11, 14]. Active TGF‐β, once released, can be deactivated by binding to the extracellular matrix or α2‐macroglobulin, among others, and can be reactivated later. Active TGF‐β remains active in the bloodstream when bound to carriers like albumin. TGF‐β associated with α2‐macroglobulin is taken up for degradation mainly by hepatic receptors and can also be excreted in urine (Fig. 1C,D) [5].

### Molecular mechanism of TGF‐β signaling

**Fig. 2:**
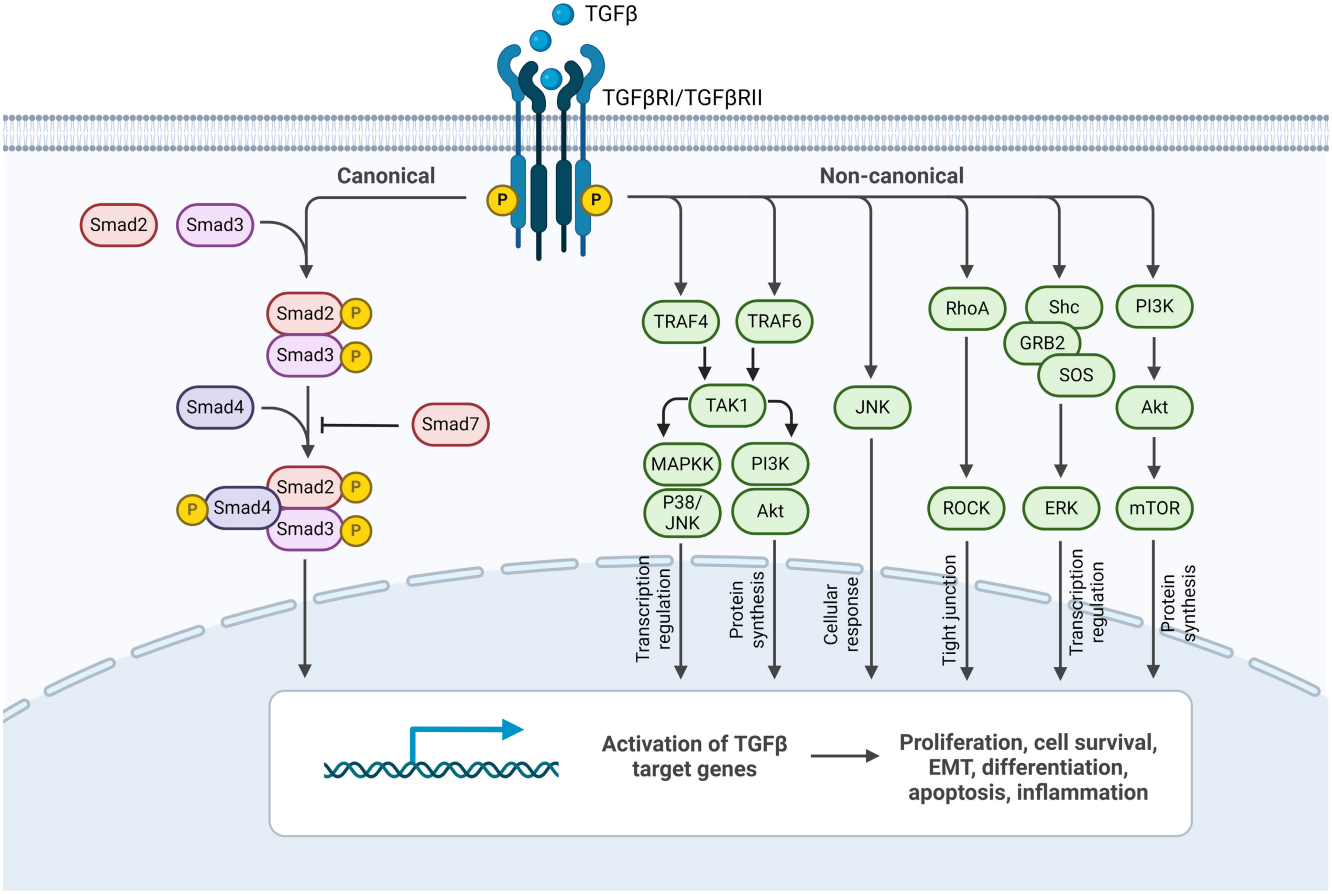
Canonical and noncanonical TGF‐β signaling pathways. Upon TGF‐β binding TGFβRII, TGFβRI is phosphorylated, which in turn either phosphorylates SMAD2/3 through a canonical pathway or phosphorylates noncanonical compartments [31]. ERKs, MAP kinases, also known as extracellular signal‐regulated kinases; GRB2, growth factor receptor‐bound protein 2; MAP3K7, mitogen‐activated protein kinase 7, also known as TAK1; MAPK, mitogen‐activated protein kinase; PI3K/AKT, phosphatidylinositol 3 kinase/protein kinase B; RhoA, Ras homolog family member A; ROCK, Rho‐associated coiled‐coil kinases; Shc, SHC‐adaptor protein; TRAF4, TNF receptor‐associated factor 4 (Created with BioRender.com).

Three TGF‐β receptor ligands (TGF‐β1, TGF‐β2, and TGF‐β3) interact with three isoforms of the TGF‐β receptor (TGFβRI, TGFβRII, and TGFβRIII), where TGFβRI and TGFβRII possess kinase activity, but TGFβRIII does not, as shown in Fig. 2 [31]. In the canonical pathway, after TGF‐β binds to its receptors, leading to the phosphorylation of TGFβRI, SMAD2 and SMAD3 are activated and phosphorylated. These phosphorylated SMAD2/3 then form complexes with SMAD4, and it is these complexes that translocate to the nucleus to regulate TGF‐β target genes [32, 33]. However, the signaling is negatively controlled by SMAD7, which promotes receptor degradation through the involvement of SMAD ubiquitination regulatory factor 1/2 (SMURF1/2) and E3 ubiquitin ligase NEDD4‐L (NEDD4‐Like), which can attenuate canonical TGF‐β signaling. The WW domain of NEDD4‐L recognizes phosphorylated SMAD2/3, which can be tagged by glycogen synthase kinase 3 (GSK3) in the nucleus, leading to the ubiquitination and subsequent degradation of the SMAD2/3 complex [34, 35].

Besides the SMAD‐mediated canonical pathway, TGF‐β binding TGFβRII can trigger the noncanonical pathway, which is SMAD‐independent. This involves recruiting like phosphatidylinositol 3 kinase (PI3K)–AKT–TOR pathway, various mitogen‐activated protein kinase (MAPK) cascades (ERK, JNK, and p38 MAPKs), and pathway downstream of Rho‐like GTPase signaling intermediates [31, 33, 36]. These kinases can modify linker regions in SMAD2/3, highlighting TGF‐β's role in numerous cellular activities, from cell growth, differentiation, apoptosis, cell motility, extracellular matrix production, angiogenesis, and cellular immune response [33]. In the PI3K/AKT pathway, AKT recruits the mammalian target of rapamycin (mTOR) upon the interaction of the TGFβRII and the TGFβRI with the p85 subunit of PI3K [37]. Meanwhile, in the MAPK pathway, transforming growth factor beta‐activated kinase 1 (TAK1) gets activated by E3 ubiquitin ligase tumor necrosis factor (TNF) receptor‐associated factors 4 and 6 (TRAF4/6), thus steering noncanonical signaling [36, 38]. TGF‐β also plays a part in Ras and the Raf–MEK–Erk MAPK signaling by influencing phosphorylation on TGFβRI and TGFβRII [36, 39]. Lastly, TGF‐β can induce RhoA and its target p160 (ROCK) in the GTPase RhoA cascade, leading to actin stress fiber formation and mesenchymal characteristics [40, 41]. Interestingly, SMAD6 negatively regulates noncanonical signaling [42].

### TGF‐β role in lung physiology and development

In healthy lungs, TGF‐β orchestrates a range of cellular responses, with specific functions driven by the interplay of the SMAD complex, master transcription factors, and their overexpressed genes [43]. This interplay is critical in guiding the differentiation and function of a diverse array of lung cell types, each playing a unique role in lung development and physiology. A pivotal player in lung morphogenesis is thyroid transcription factor‐1 (NKX2‐1) expressed in pulmonary epithelial cells, crucial for various lung developmental processes, including promoting lung‐specific gene transactivation, such as the alveolar epithelial cell‐specific surfactant genes (SpA, SpB, and SpC) as well as the bronchiolar exocrine cell‐specific gene CC10 [43, 44]. The differentiation and roles of these cell types, from epithelial to mesenchymal cells, underscore the complex orchestration of signals that guide lung organogenesis and functional maturation [45]. However, it is important to note that while studies using lung cancer cell lines, such as A549 adenocarcinoma cells, provide invaluable insights into the pathological roles of EMT, they may not clearly inform about the role of EMT in normal lung development and physiology. This distinction underscores the need for caution when extrapolating findings from cancer research to understand normal lung functions [46]. NKX2‐1 fortifies cell–cell junction by upregulating proteins such as E‐Cadherin and repressing EMT, thereby modulating lung adenocarcinoma cell behavior in conjunction with TGFβ [47]. Notably, TGF‐β represses the transcription of pulmonary surfactant protein‐B (SpB) through its interaction with NKX2‐1, impacting pulmonary tissue homeostasis [48]. Furthermore, during lung development, TGF‐β isoforms are pivotal in lung organogenesis, influencing processes like branching morphogenesis, alveolarization, and cell differentiation through interaction with various signaling mechanisms, including fibroblast growth factor, sonic hedgehog (SHH), Wnt/β‐catenin, and BMP [9, 49, 50]. Different TGF‐β isoforms localize in specific areas, like TGF‐β1 near branching points, TGF‐β2 in the distal epithelium, and TGF‐β3 in proximal mesenchymal cells. As we examine the regulatory role of TGF‐β in lung, it is important to clarify the context within which EMT is considered. Although EMT is a fundamental process in developmental biology, implicating its occurrence in normal lung development or adult lung physiology requires nuanced understanding. The well‐established significance of EMT in pathological states such as lung cancer, where it underpins metastasis and tumor invasiveness, contrasts with its speculative role in the healthy lung. This highlights the necessity for clear delineation in studies and discussions of EMT, ensuring distinctions are made between its documented pathological roles and less defined contributions to normal lung physiology. This highlights the necessity for clear delineation in studies and discussions of EMT, ensuring distinctions are made between its documented pathological roles and less defined contributions to normal lung physiology [51].

**Fig. 3:**
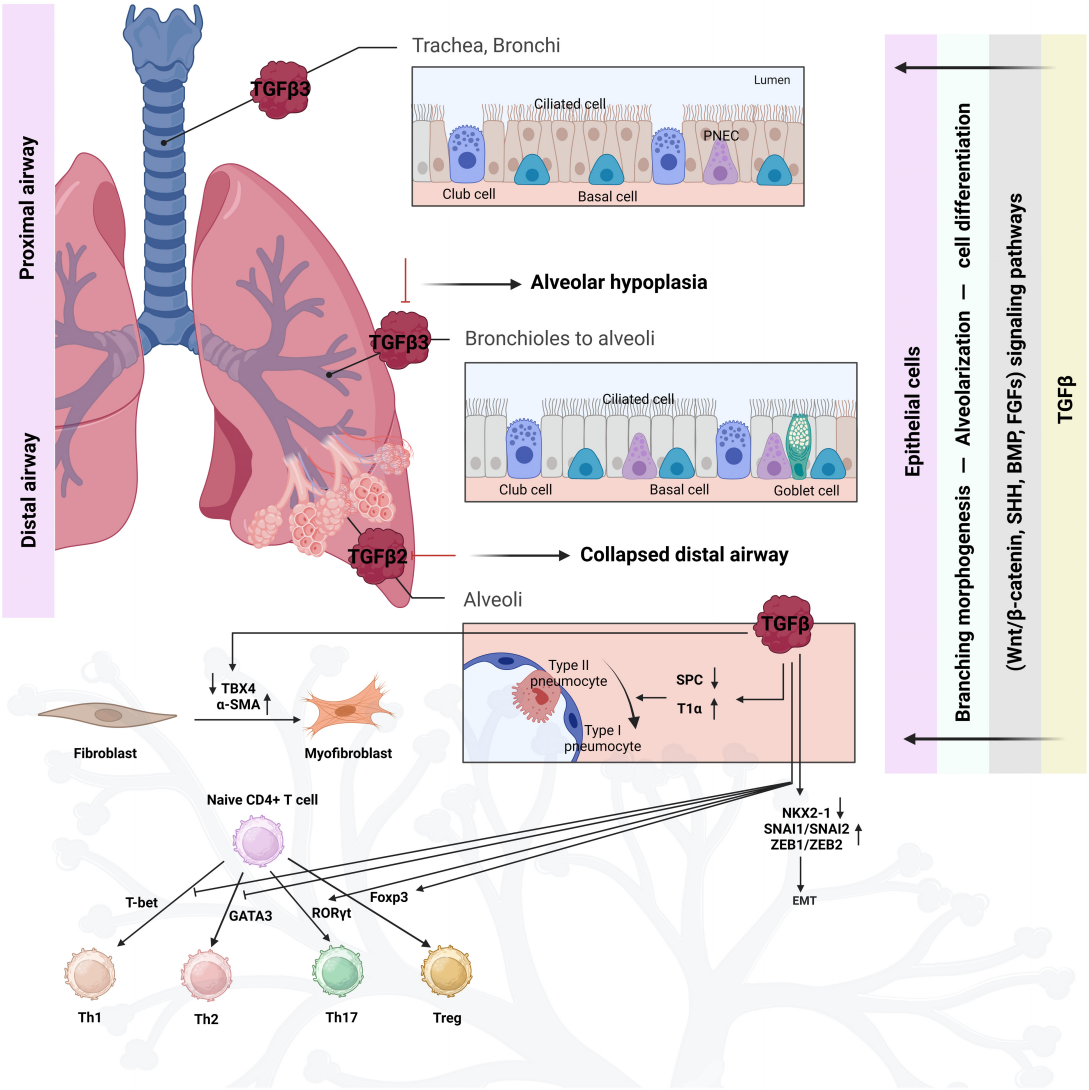
Cell‐specific function of TGF‐β. Myofibroblasts are specialized cells that have a vital function in the process of wound healing and tissue repair. Within the lungs, myofibroblasts undergo differentiation from fibroblasts, which are a more prevalent kind of cell, in reaction to numerous stimuli such as damage, inflammation, and fibrosis. The differentiation process is closely controlled by an intricate interaction between signaling pathways and transcription factors [65]. The lining of the conducting airways mostly consists of basal, club, goblet, and ciliated cells. Among these, the most significant cells engaged in the process of EMT are responsible for producing myofibroblasts. In addition to them, there are also additional epithelial cells present in the airways, albeit they are present in a minimal percentage. However, they include PNECs, which function as stem cells and have been implicated in stimulating the growth and movement of fibroblasts [66, 67]. TGF‐β induces EMT in alveolar epithelial cells, converting them to mesenchymal cells. EMT‐related transcriptional repressors (SNAI1/SNAI2 and ZEB1/ZEB2) are induced, and NKX2‐1 is repressed by TGF‐β, which promotes EMT (increases cell motility and decreases cell polarity). TGFβ induces differentiation of type II alveolar epithelial cells to type I alveolar epithelial cells by repressing transcription SPC expression and T1α. TGF‐β induces fibroblast differentiation into myofibroblasts by repressing TBX4 and upregulating α‐SMA (increases contraction and ECM production). In naïve CD4^+^ T cells, TGFβ inhibits the derivation of Th1 and Th2 cells. At the same time, it promotes the formation of Th17 and T regulatory cells (Treg) by regulating their corresponding master transcription factor [9]. CD4^+^ T helper cells undergo differentiation into three distinct subtypes known as Th1, Th2, and Th17 cells. These subtypes are distinguished by the specific cytokines they produce, namely IFN‐γ, IL‐4, and IL‐17, respectively [68]. TGF‐β, in conjunction with IL‐6, is recognized for stimulating the production of Th17 cells. These cells attract neutrophils to the airway and promote eosinophilic inflammation mediated by Th2 cells [9, 69]. On the other hand, TGF‐β hinders the development of Th1 and Th2 cells by suppressing the activity of T‐bet and GATA3 transcription factors, respectively [70]. SPC, surfactant protein C; T1α, type I alveolar epithelial cells express podoplanin; TBX4, T‐box transcription factor; Th, T helper; Treg, regulatory T; α‐SMA, α‐smooth muscle actin (Created with BioRender.com).

Table 1 summarizes the distinct roles of TGF‐β signaling in lung development using genetically engineered mouse models [9]. Deficiency in various components of the TGF‐β1 signaling pathways led to specific lung abnormalities [52, 61]; TGF‐β2 deficiency leads to collapsed distal airways [54]; TGF‐β3 knockout mice showed alveolar hypoplasia [55]; TGFβRII‐deficient mice demonstrated abnormal alveolarization and emphysema [62]; and TGFβRI deficiency mouse had immature alveoli and reduced club cell population [59]. SMAD3‐knockout mice developed impairment in alveolarization and centrilobular emphysema [53, 56]. Additionally, mesodermal deletion of TGFβRII intercepted lung branching, causing cystic adenomatoid malformation [58], while its deletion in TGFβRI impacted mesodermal progenitor cells, leading to pulmonary hypoplasia [60]. Lung epithelial morphogenesis and differentiation can be interrupted by ectopic expression of TGF‐β1 [63]. External TGF‐β1 application inhibited lung branching [64]. Overall, these studies emphasize the importance of TGF‐β in lung epithelial growth and branching morphogenesis (Fig. 3) [9].

**Table 1:** The role of TGFβ signaling in lung organogenesis was investigated by mouse models [9].

Mouse model	Phenotype in the lungs	Phenotype in other organs	Reference
TGF‐β1 (−/−)	Perivasculitis; interstitial pneumonia	Systemic inflammation	[52, 53]
TGF‐β2 (−/−)	Collapsed distal airways with dilated conducting airways	Cardiac, craniofacial, limb, spinal column, eye, inner ear, and urogenital defects	[54]
TGF‐β3 (−/−)	Atelectatic, pseudoglandular histology with alveolar hypoplasia; mesenchymal thickening; extensive intrapulmonary and pleural hemorrhage; dilated conducting airways	Cleft palate	[55]
SMAD (−/−)	Progressive lung airspace enlargement and emphysematous changes; reduced pulmonary alveolarization and subsequent centrilobular emphysema	Defects in immune function with inflammatory lesions; decreased growth rate	[53, 56]
TGFβRII (−/−)	Abnormal lung branching and reduced cell proliferation	Defective secondary ventral body wall formation, congenital diaphragmatic hernia, and abnormal cardiac development	[57]
TGFβRII (−/−)	Failure in branching morphogenesis and cystic airway malformations	Abnormalities in multiple organs	[58]
TGFβRI (−/−)	Immature alveoli and formation of a disorganized and multi‐layered epithelium in the proximal airways; marked reduction in the number of club cells		[59]
TGFβRI (−/−)	Disrupted differentiation of mesodermal progenitor cells, resulting in the formation of pulmonary hypoplasia		[60]

Alveolar epithelial type I cells facilitate gas exchange, whereas type II offers protection. Figure 3 [9] illustrates that both types (I and II) connect the alveoli to airway epithelial cells, including basal, secretory, ciliated, and neuroendocrine cells [71]. TGF‐β inhibits epithelial cell growth and affects neonatal type II alveolar cell proliferation [72]. It also drives EMT, enhancing cell mobility and invasiveness by disrupting cell junction and recruiting transcriptional repressors, downregulating junction proteins and NKX2–1 (Fig. 3) [9, 73]. *In vitro* studies have shown that TGF‐β represses alveolar epithelial type II markers, aiding the differentiating of type II cells into type I cells, essential for alveolar homeostasis [9, 74, 75]. Conversely, inhibiting TGF‐β signaling promotes the transition from type I to type II cells [76]. Therefore, TGF‐β has a vital pleiotropic role in alveolar epithelial cell growth suppression, EMT, and reciprocal differentiation between type I and II.

### TGF‐β in lung cancer

**Fig. 4:**
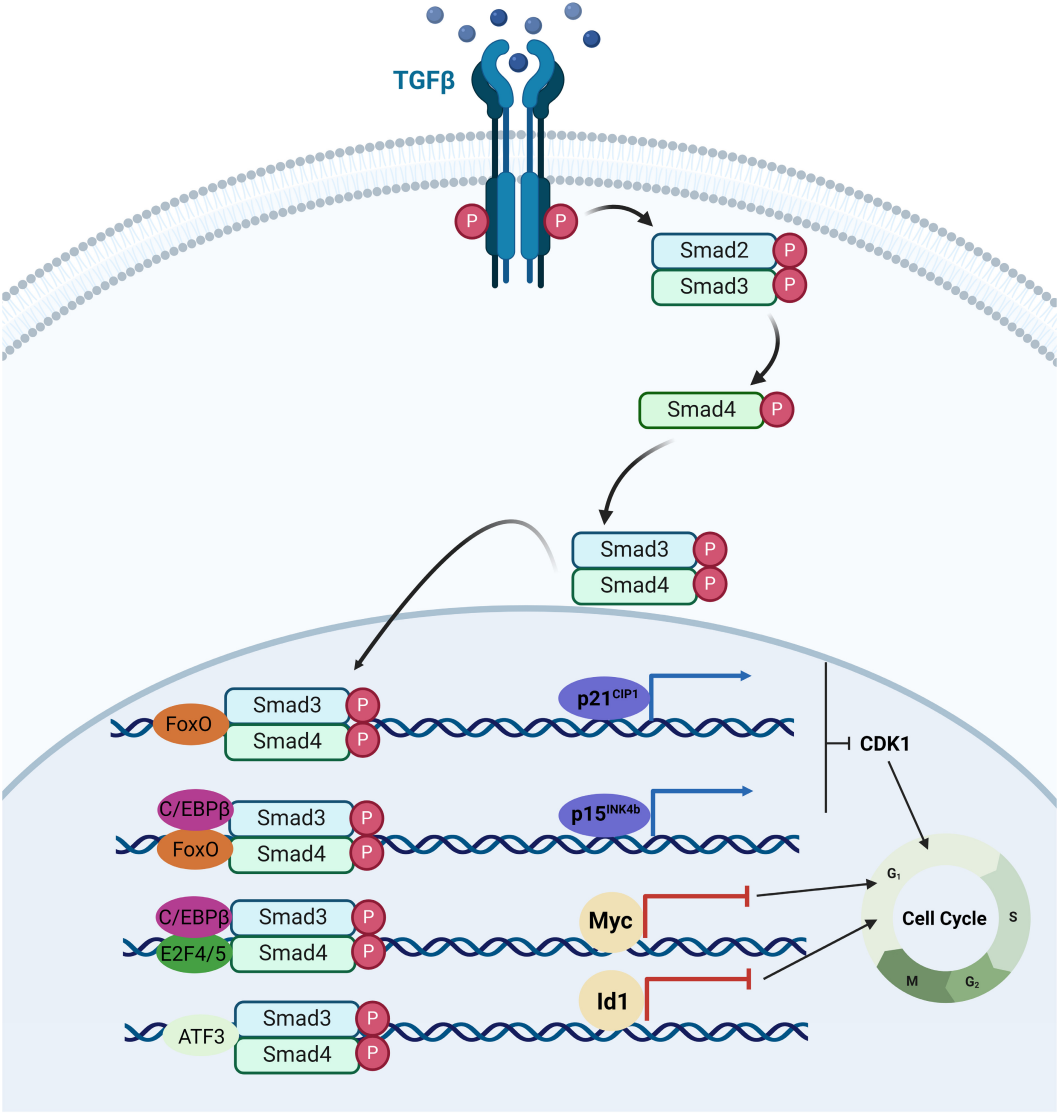
Cell growth arrest mechanisms induced by TGF‐β in epithelial cells [57]. C/EBPs, CCAAT/enhancer‐binding proteins; ATF3, activating transcription factor 3; E2F4/5, E2F transcription factor 4 (Created with BioRender.com).

TGF‐β plays a multifaceted role in carcinogenesis. Initially, it functions as a tumor suppressor by inhibiting cell proliferation, promoting differentiation, and inducing apoptosis in normal cells. However, this role reverses in advanced cancer stages, leading to tumor promotion and metastasis due to increased expression and signaling activation [77]. This shift involves both canonical (SMAD‐dependent) and noncanonical pathways. The canonical TGF‐β pathway promotes lung metastasis via activation of angiopoietin‐like 4 (ANGPTL4) [78]. Additionally, mutations in genes encoding TGFβRI and TGFβRII have been identified in various cancers, possibly contributing to tumor progression [77, 79, 80]. TGF‐β inhibits cell proliferation by regulating the cell cycle, involving mechanisms like the induction of CDK inhibitors p21Cip1 and p15Ink4b and suppressing proliferative factors like c‐Myc (Fig. 4) [81, 82, 83, 84, 85]. This inhibition is context‐specific, often observed in cells under stress or injury. The repression of c‐Myc by the SMAD3–SMAD4 complex, which involves various transcription factors, underscores TGF‐β's role in cell cycle arrest [83, 84, 86]. Noncanonical TGF‐β signaling also plays a part in cell‐growth inhibition [87].

TGF‐β signaling can influence apoptosis based on cell type and physiological and environmental contexts, with both canonical SMAD‐dependent and noncanonical SMAD‐independent pathways potentially suppressing cancer growth and promoting tissue homeostasis. The specifics of TGFβ‐induced apoptosis in epithelial tissues remain unclear [88], but various factors have been identified that connect TGF‐β signaling to apoptosis across different cell lines. Noncanonical TGFβ‐induced apoptosis involves adaptor protein DAXX, promoting JNK activation [89] and activation via the TRAF6–TAK1–JNK/p38 pathway [90, 91]. The tumor suppressor p53, p38 MAPK, and SMADs collectively contribute to TGF‐β's proapoptotic function [92].

TGF‐β directly contributes to growth arrest and homeostasis and represses tumorigenesis by regulating paracrine factor production in the tumor stroma [82]. Overexpression of kinase‐deficient TGFβRII in mouse mammary stroma upregulates the fibroblasts' hepatocyte growth factor (HGF), promoting epithelial branching [83, 84, 85]. Further, *in vivo* studies showed that TGFβRII‐deficient fibroblasts increase HGF expression, affecting neighboring epithelial cells and leading to the development of certain carcinomas [88, 93, 94]. Upon tumorigenesis, TGF‐β suppresses immune and inflammatory responses [83, 86], affecting various cells like CD8^+^ cytotoxic T cells, CD4^+^ T cells, macrophages, dendritic cells, and NK cells. In contrast, the production of regulatory T cells and Th17 cells is induced by TGFβ [95, 96]. TGFβ signaling plays a multifaceted role in cancer progression. While it promotes cytostasis and apoptosis in normal cells, mutations in its tumor‐suppressive arm can enhance tumorigenesis and cancer progression.

**Fig. 5:**
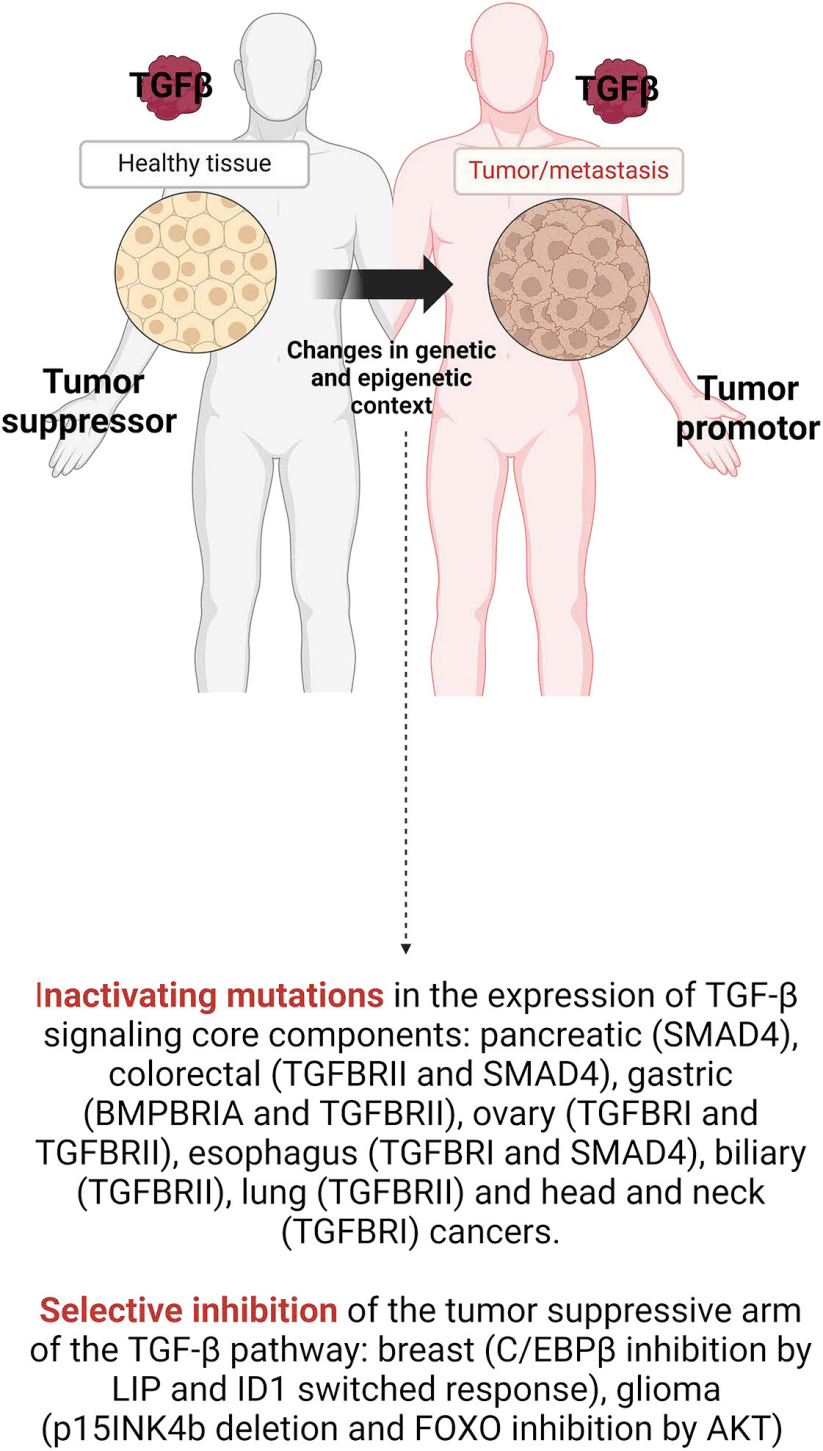
Inactivation of TGFβ core components vs inhibition of downstream targets. Inactivating mutations in the expression of TGFβ signaling core components, such as the receptors and the SMAD transcription factors. Selective inhibition of the tumor suppressive arm of the TGFβ pathway [99]. DAPK, death‐associated protein kinase 1; DAXX, death‐associated protein 6; GADD45B, growth arrest and DNA damage‐inducible beta (Created with BioRender.com).

Genetic changes in tumor cells help them to escape from tumor suppressive activity of TGF‐β. Consequently, higher TGF‐β activity can paradoxically promote tumor progression and metastatic dissemination via various mechanisms, including EMT, immune system evasion, and tumor microenvironment regulation [87, 97, 98]. These mechanisms show the vital role of TGF‐β in promoting tumor progression and invasiveness that eventually leads to metastasis. TGF‐β is essential in promoting cancer cell migration and invasiveness by facilitating the EMT, a process where epithelial cells acquire fibroblastic properties [99]. TGF‐β is a potent inducer of EMT, as it has been found in embryonic development [100]. Both canonical and noncanonical TGF‐β signaling pathways are involved in the induction of EMT [99]. Loss of E‐cadherin promotes TGFβ‐induced EMT in cancers, whereas its overexpression represses the invasion of cancer cells [93, 101]. The E‐cadherin repression was mediated by zinc‐finger transcription factors and SMAD‐dependent expression of high mobility group A2 (HMGA2) [102]. Also, it has been found that cell–cell junctions can be dissociated by TGFβRII‐induced phosphorylation of Polarity Protein Par6 (Fig. 5) [103].

Recent investigations provide clarity on this intricate connection. A meta‐analysis highlighted a strong correlation between elevated TGF‐β expression and poor survival outcomes in lung cancer patients, bringing to the fore its critical role in disease pathophysiology [104]. Delving into molecular crosstalk, evidence points to an interesting interplay between TGFβ and Ras signaling pathways. Notably, the Jumonji domain containing‐3 (JMJD3) enhances the effect of TGF‐β in activating pathways like SMAD that lead to EMT in Ras‐activated lung cancer cells, which exemplifies the convergence of diverse signaling events in tumor progression [105]. In parallel, studies on mouse lung adenocarcinoma cell lines underscore the capacity of TGF‐β capacity to induce EMT, emphasizing its promotive role in lung adenocarcinoma [89].

The way TGF‐β interacts with the lung cancer environment is crucial. Research indicates that the onset of lung cancer is influenced by genetic changes and environmental factors such as smoking and chronic inflammation. In this context, TGF‐β plays a major role in promoting cancer invasiveness and the ability to spread, mainly by initiating EMT. This relationship between TGF‐β and EMT could serve as a marker to predict how lung cancer will progress and to identify effective treatments that might stop cancer spread in its early stages, as seen in some animal studies [90]. Moreover, within the hypoxic tumor microenvironment, TGF‐β has been shown to induce a deep senescent state marked by a unique 14‐gene SASP (senescence‐associated secretory phenotype). When left unchecked, this phenomenon seems to play a role in fostering an immune‐suppressive phenotype, which could hinder the efficacy of immune therapies [91]. In another compelling study, the acidic tumor microenvironment was shown to enhance lung cancer metastasis by inhibiting miR‐7‐5p, leading to increased TGF‐β2 expression. This pivotal discovery of the miR‐7/TGF‐β2 axis in acidic pH‐mediated lung cancer progression introduces miR‐7 as a potential therapeutic target and reliable biomarker for non‐small‐cell lung cancer (NSCLC) [92].

## TGF‐β and immune cells: an essential regulating axis in the tumor microenvironment and development

TGF‐β is essential for cancer immune control, inhibiting the immune system's response, encouraging tumor development, and enabling cancer cell immune evasion. TGF‐β signaling abnormalities cause inflammation and tumors. Understanding TGF‐β regulation at the tumor–host immunity interface may aid in creating effective TGF‐β antagonists and biomarkers for patient selection and therapy effectiveness. Transitioning to the sphere of immunology, it becomes evident that the influence of TGF‐β spans beyond traditional cellular pathways, impacting immune responses within the lung cancer context. The following segments will delve into this facet, elucidating the profound interplay between TGF‐β and immune dynamics in lung cancer.

### Immune evasion

Upon tumor onset, the immune system recruits T cells and natural killer (NK) cells to identify and destroy cancer cells. However, TGF‐β in those cancer cells that already bypassed the TGFβ‐induced tumor suppression promotes the progression of cancer cells by helping them to escape from the immune system surveillance [106].

The T‐cell activation process is suppressed by TGFβ‐induced inhibition of antigen‐presenting cells (APCs) like dendritic cells responsible for T‐cell stimulation [99, 107]. It was shown that transfecting highly immunogenic murine tumors with TGF‐β1 cDNA helped tumor cells to escape immune surveillance significantly [108]. Another mouse model study showed that inhibition of TGF‐β signaling in T lymphocytes through transgenic expression of the dominant negative form of TGFβRII (DN‐TGFβRII) in these cells repressed growth and metastasis in this mouse model [82, 109]. This study found that T‐helper (CD4^+^) and cytolytic T cells (CTL or CD8^+^) were involved in defending against tumor cells. Another study also found that CD4^+^CD25^+^ regulatory T cells repressed the activity of NK cells by producing higher TGF‐β [110].

### Adaptive immunity

#### TGF‐β inhibits T helper 1 and cytotoxic T‐cell responses

*T helper 1* (Th1) immune responses against cancer are one of the most prominent T‐cell responses, Naïve T cells cannot be differentiated from Th1 cells in the presence of TGF‐β [111]. Conversely, TGFβRII inhibition on T cells increases Th1 differentiation and immune responses [112, 113]. TGF‐β blocks Ca^2+^ influx and dampens CD4^+^ T‐cell activation in early stage inhibiting the development of T‐bet and GATA‐3 transcription factors and limiting differentiation of Th1 and Th2 lineages, respectively [114]. Consistent with these findings, TGF‐β inhibits Th1 differentiation through the suppressed expression of interleukin 12 receptor, beta 2 subunit interleukin 12 receptor, beta 2 subunit (IL‐12Rβ2), and ectopic expression of T‐bet into developing Th1 cells abolishes the inhibitory effect of TGF‐β [115]. TGF‐β limits the induction of interferon gamma IFN‐γ‐producing Th1 cells through decreasing *Signal transducer and activator of transcription 4* (STAT4) [116]. TGF‐β1 through SMAD3‐dependent and SMAD3‐independent pathways also inhibits T‐cell proliferation [117].

Suppression of CXCR3 ligands and decreased CD8^+^ T‐cell trafficking to the tumor have been shown in mice melanoma lung metastasis model as an immune escape mechanism [118]. TGF‐β suppresses chemokine receptor CXCR3 transcription by SMAD2/3 and thereby limiting CD8^+^ T‐cell trafficking to the tumor. Studies on the impact of CXCR3 ligand in IL‐7/IL‐7Rα‐Fc–mediated antitumor activity revealed that when treating mice with lung cancer using IL‐7/IL‐7Rα‐Fc, it raised the levels of chemokine ligands CXCL9 and CXCL10, IFNγ, and IL‐12, but it reduced the levels of IL‐10 and TGF‐β [119]. It has also been found that CXCR3 is expressed in lung adenocarcinoma cell lines, which suggests CCR7 and CXCR3 play a role in lung adenocarcinoma metastasis to lymph nodes [119].

**Fig. 6:**
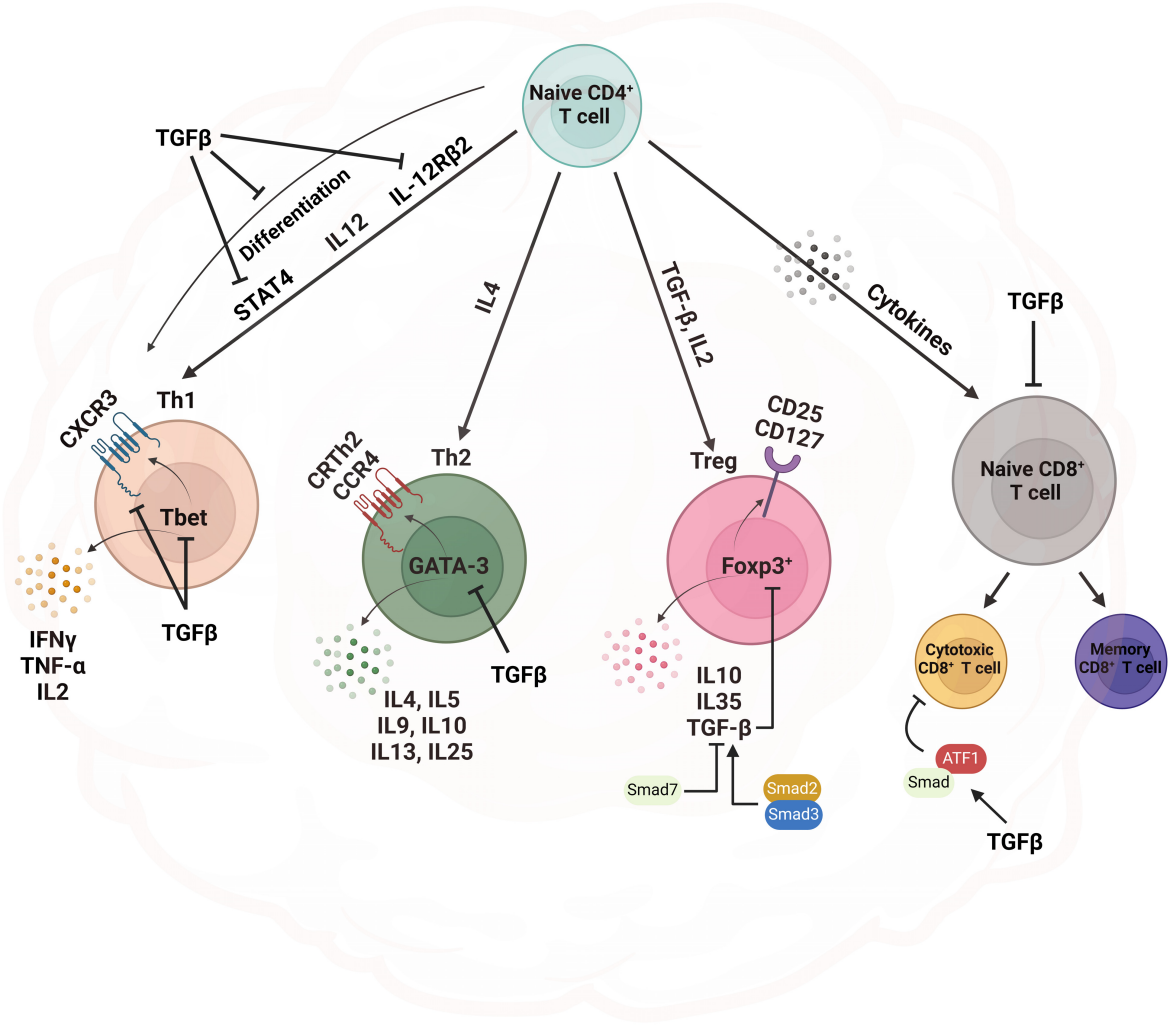
Regulation of adaptive immunity by TGF‐β. CD4^+^ T cells play a crucial role in orchestrating adaptive immune responses by releasing cytokines and engaging in direct cell‐to‐cell interactions. In contrast, CD8^+^ T cells are primarily devoted to differentiating into cytotoxic effector cells. Antigen‐presenting cells (APCs) are responsible for the recognition of tumor antigens. Upon activation by antigen‐presenting cells (APCs), naïve CD4^+^ T cells undergo differentiation into distinct subpopulations, including T helper (Th)1, Th2, Th9, Th17, Th22, T follicular helper (Tfh), and T regulatory cells (Treg), in response to certain cytokine‐polarized settings. This differentiation process is mediated by the engagement of T‐cell receptors (TCR) on the surface of naïve CD4^+^ T cells [129, 130]. TGF‐β plays an integral part in controlling adaptive immunity by exerting inhibitory effects on the differentiation of Th1 and Th2 lineages. Furthermore, it can impede the cytotoxic T‐cell response and hinder the progression of T‐bet and GATA‐3, which are transcription factors that play a key role in T‐cell differentiation. Consequently, this might result in a decrease in the production of cytokines that are typically released by Th1 and Th2 cells. Likewise, it has been shown that TGF‐β can inhibit the expression of CXCR3, a chemokine receptor protein found on Th1 cells, which leads to immune evasion. Tregs are essential in maintaining immunological homeostasis. Concerning Tregs, the suppressor CD4^+^ T‐cell phenotype is induced by TGF‐β via numerous routes. The transcriptional downregulation of SMAD7 by Foxp3^+^ plays a vital part in supporting the acquisition of Tregs traits. SMAD7, a significant negative regulator of TGF‐β production, is suppressed by Foxp3^+^ at the transcriptional level. Furthermore, the deletion of SMAD2and SMAD3, which are known to be positive regulators of TGF‐β production, in T cells led to the depletion of Foxp3^+^ cells and the inhibition of Th1 differentiation. CRTH2, chemoattractant receptor‐homologous molecule expressed on Th2 cells; CXCR3, C‐X‐C motif chemokine receptor 3; Foxp3^+^, forkhead box P3, also known as scurfin; IFN‐γ, interferon gamma; IL, interleukin; IL‐12Rβ2, interleukin 12 receptor subunit beta 2; STAT4, signal transducer and activator of transcription 4; T‐bet, T‐box transcription factor TBX21, also called T‐bet; TGF‐β, transforming growth factor beta; TNF‐α, tumor necrosis factor α (Created with BioRender.com).

TGFβRII‐deficient CD4^+^ and CD8^+^ cells increase T‐cell receptor (TCR)‐dependent activation upon weak antigen stimulation and promote cytotoxic effects through increasing granzyme and perforin expressions [112, 120]. TGF‐β‐activated SMADs and cyclic AMP‐dependent transcription factor 1 (ATF‐1) transcriptional factors suppress CD8^+^ cytotoxic functions *in vivo* via promoter repression of granzyme B and IFN‐γ [107]. TGF‐β induces cell cycle arrest in T cells [121] and apoptosis of effector T cells via downregulation of Bcl‐2 [122] and upregulation of pro‐apoptotic Bcl‐2‐family protein BIM [123]. Also, TGF‐β downregulates the expression of major histocompatibility complex (MHC) class II molecules by suppressing class II transactivator (CIITA) expression, thus impairing antigen presentation and CD4^+^ T‐cell priming [124, 125, 126]. TGFβ1‐mediated SMAD3 suppresses antigen‐specific T cells *in vitro* and TILs *in vivo* through PD‐1 upregulation [127]. Systemic TGF‐β blockade in the host can lead to reduced cytostatic effects of TGF‐β on tumor cells [128], but targeted blockade of TGF‐β signaling in T cells may provide a better strategy to enhance antitumor immunity and eradicate lung cancer (Fig. 6).

#### TGF‐β induces T regulatory phenotype

In healthy tissues, T regulatory cells (Tregs) are in low frequency and suppress effector T‐cell functions to maintain hemostasis. However, in many cancers, their frequencies are increased, which enforces tolerance to tumor antigens and facilitates tumor immune evasion [52, 61, 131, 132]. The peripheral CD4^+^ T cells undergoing suboptimal stimulation could produce TGF‐β, promoting differentiation to the Treg stage [133]. TGF‐β induces suppressor CD4^+^ T‐cell phenotype through different mechanisms. They induce Foxp3^+^ Tregs expression from naive peripheral CD4^+^CD25^−^ T cells, which mediate their transition into CD4^+^CD25^+^ suppressor T cells. This inhibits CD4^+^ T‐cell proliferation and Th1 and Th2 cytokine production *in vitro* [134]. SMAD7, the critical negative regulator of TGF‐β expression is downregulated by Foxp3^+^ at the transcriptional level, promoting the acquisition of Tregs properties [33]. Consistent with these findings, conditional knockout of SMAD2 and SMAD3, which are positive regulators of TGF‐β expression, led to the loss of Foxp3^+^ mediated by TGF‐β and suppression of Th1 development in T cells [135].

Limited studies have investigated the role of TGF‐β in developing T regulatory phenotype in lung cancer. Baratelli *et al*. [136] found that in patients with NSCLC, TGF‐β can induce competent Treg from human peripheral blood lymphocytes through a mechanism that depends on prostaglandin‐endoperoxide synthase 2/prostaglandin E2 (COX‐2/PGE_2_) signaling. They found that TGF‐β stimulated PGE_2_ production and COX‐2 expression in both peripheral blood lymphocytes and purified CD4^+^ T cells. Pharmacologic inhibition of COX‐2 suppressed the TGFβ‐induced PGE_2_ production.

CD25^+^CD4^+^ T cells contribute to the control of memory CD8^+^ T cells, affecting the immune responses against cancer [137]. Elucidating molecular mechanisms of TGF‐β‐driven transition of naïve T cells toward regulatory T‐cell phenotypes, and investigating new physiological inducers of Foxp3^+^ expression in CD4^+^ naïve or responder T cells, will enable the manipulation of specific populations of regulatory T cells in lung cancer. Moreover, in a pro‐inflammatory cytokine environment, IL‐21, synergizing with IL‐2, enhances T‐cell proliferation, subsequently counteracting IL‐2/TGFβ‐induced regulatory T‐cell development, favoring differentiation of effector CD4^+^ T cells [138]. IL‐21 and IL‐21R expression are decreased in NSCLC patients, and IL‐21 treatment regresses tumor growth and invasion in these patients [139]. Although the relationship between IL‐21 and TGF‐β is unclear, this treatment might provide a molecular target for lung cancer therapy.

The inhibition of TGF‐β and vascular endothelial growth factor (VEGF) signaling pathways synergistically reduces Tregs, increases infiltrating T cells, and restores tumor sensitivity to anti‐PD‐1 and anti‐CTLA‐4 treatments, eradicating immunogenic tumors [140]. A recent clinical study found that high frequencies of circulating Treg cells 1 week after immunotherapy was associated with a favorable clinical response in NSCLC patients treated with anti‐PD‐1 immunotherapy (nivolumab or pembrolizumab). Accordingly, high expression of TGF‐β was correlated with high levels of Treg cells and is associated with favorable clinical outcomes [141]. TGF‐β is a very potent immunosuppressant; long‐term systematic blockage of TGF‐β may result in autoimmunity in patients; therefore, it is critical to find the delicate balance between tumor immunity and autoimmunity maintained by Tregs cells in treating tumors.

### Innate immunity

#### TGF‐β suppresses natural killer cells

**Fig. 7:**
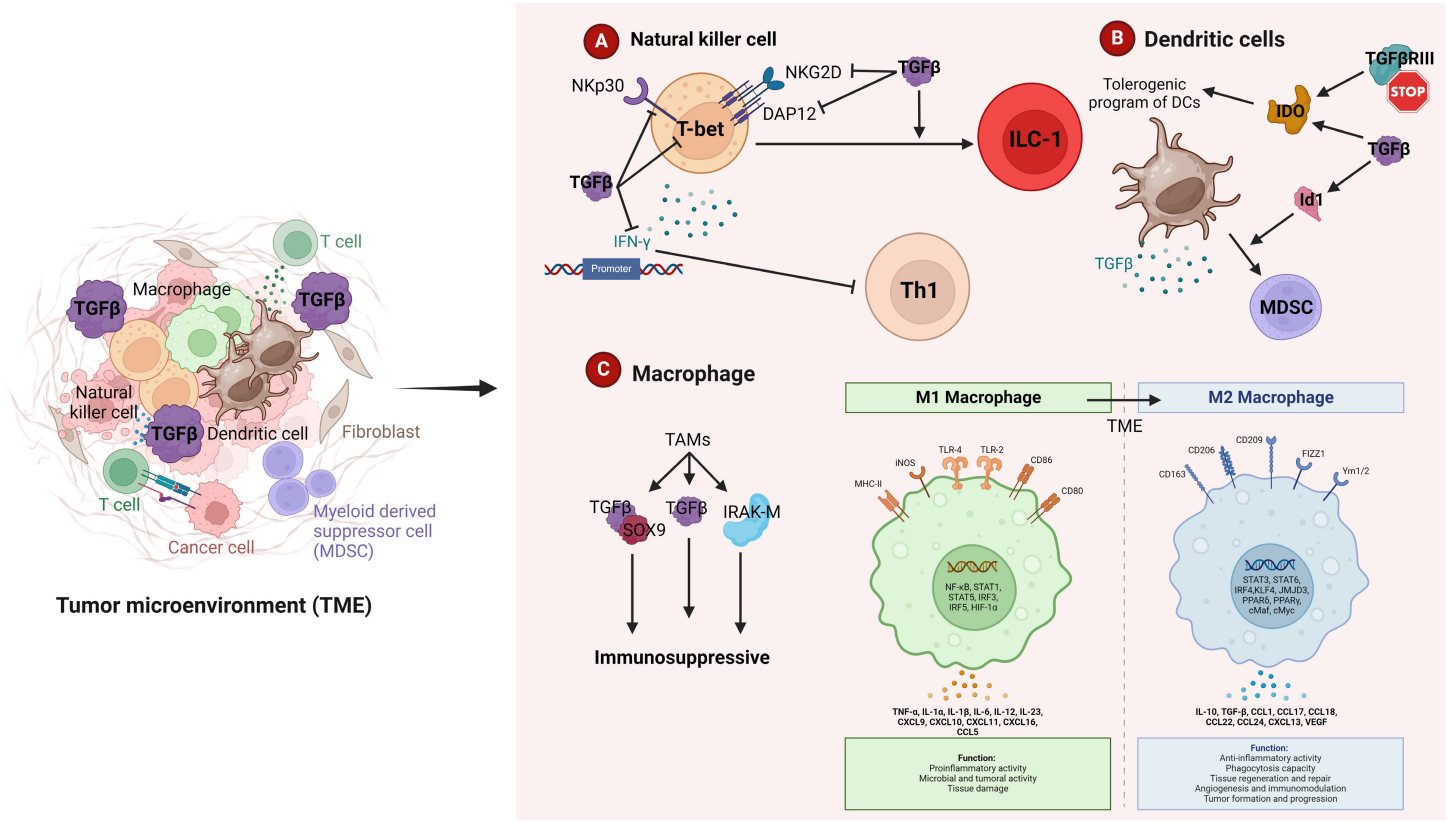
Regulation of innate immunity by TGF‐β. Innate immune cells comprising NKs, DCs, and macrophages are critical immune system components. TGF‐β influences the innate immune system by inhibiting NK cells and regulating DCs and macrophages, forming a negative immune regulatory network. (A) TGF‐β suppresses NK cells in different ways. It limits Th1 immune responses by suppressing NK cell production of T‐bet and IFN‐γ (via SMAD2, 3, 4, and T‐bet‐independent negative regulatory action on the IFN‐γ promoter). Additionally, NKG2D and NKp30 expression are suppressed by TGF‐β. Finally, TGF‐β induces ILC‐1 differentiation of NK cells. (B) DCs regulate Th1 and Treg immune responses by the TGF‐β signaling pathway. Tumor‐derived TGF‐β promotes tumor progression and metastasis by switching DCs into MDSC. Additionally, TGFβ maintains IDO activation in DCs, conferring a tolerogenic program. Cancer Treg cell infiltration is facilitated by the loss of TGFβIII by upregulating IDO in plasmacytoid DCs and the CCL22 chemokine in myeloid DCs. (C) TAM generates TGF‐β as one of the key immunosuppressive cytokines, and it is well recognized that TME polarizes macrophages toward the M2 phenotype, a phenotype with immunosuppressive properties. TAMs express a potent negative regulator of TLR signaling called IRAK‐M at much greater levels than peritoneal macrophages. In NSCLC clinical samples, TAMs also enhance tumor metastasis through the TGF‐/SOX9 axis. (IRAK)‐M, interleukin receptor‐associated kinase; DCs, dendritic cells; Id1, inhibitor of differentiation 1; IDO, indoleamine 2,3‐dioxygenase; ILC‐1, type 1 innate lymphoid cells; MDSCs, myeloid‐derived suppressor cells; NK, natural killer cells; NKG2D, natural killer group 2, member D; DAP12, also known as TYROBP and KARAP; TAM, tumor‐associated macrophages; Th, T helper (Created with BioRender.com).

Natural killer (NK) cells are critical components of innate immune cells that respond rapidly to tumor cells by their cytotoxic functions [142]. It has been found that TGF‐β suppresses NK cell functions through different mechanisms. TGF‐β downregulates T‐bet and IFN‐γ expression on NK cells via SMAD 2, 3, 4, and T‐bet independent negative regulatory effect on IFN‐γ promoter, thus restricting Th1 immune responses [143, 144]. TGF‐β inhibits natural killer group 2D (NKG2D) receptor and natural cytotoxicity receptor 3 (NKp30) expression that mediate recognition of malignant transformed cells [145]. Impaired NK cytotoxicity due to the correlation between TGF‐β and NKG2D receptors has been shown in lung cancer [146, 147]. The transcriptional levels of DAP12, an adaptor of NK cytotoxic receptors including NKG2D, are repressed by TGFβ‐induced miR‐183 in lung cancer [148]. TGF‐β disrupts mTOR signaling downstream of stimulatory cytokines such as IL‐15, suppressing NK cell activity and function [149]. TGF‐β converts NK cells into intermediate type 1 innate lymphoid cells (ILC‐1) *in vitro* and *in vivo*, suggesting TGFβ‐mediated NK plasticity and tumor escape by innate immunity [150, 151]. It has also been found that TGF‐β1 did not affect the perforin or Fas‐ligand expression, implying that TGF‐β1 could suppress NK cell activity mainly through NKG2D modulation, which is not associated with alteration of lytic moieties. Modulating SMAD/DAP12 gene expression and targeting NK cells to augment IFN‐γ response during antigen challenge could be a targeted therapy for lung cancer treatment (Fig. 7A) [152].

#### TGF‐β subverts dendritic cell function

Dendritic cells (DC) are potent antigen‐presenting cells and play crucial roles in tumor immunity. DCs are essential in regulating Th1 and Treg immune responses by the TGF‐β signaling pathway [153]. Under tumor microenvironment in lung carcinoma condition, DCs are instructed to secrete TGF‐β, followed by promoting the acquisition of Treg phenotype by CD4^+^ T cells. This suggests an altered function of DCs by tumor cells to produce TGF‐β, contributing to immune evasion [154, 155]. Tumor‐derived TGF‐β switches DC differentiation to myeloid‐derived suppressor cells (MDSCs) though upregulating inhibitor of differentiation 1 (Id1), DNA binding inhibitor, promoting tumor progression and metastasis [156]. Additionally, TGF‐β confers a tolerogenic program of DCs by sustaining the activation of immunosuppressive molecules indoleamine 2,3‐dioxygenase (IDO) [157, 158]. As a suppressor role of a TGFβRIII in cancer progression and metastasis, it has been shown that loss of TGFβRIII mediates immune evasion by upregulating IDO in plasmacytoid DCs and the CCL22 chemokine in myeloid DCs. Identifying new master transcriptional regulators of myeloid cell differentiation and investigating the role of known interferon regulatory factor 8 (IRF8), transcriptional factor for DC differentiation, can help control systemic tumor‐induced immunosuppression in lung cancer (Fig. 7B).

#### TGF‐β regulates macrophage phenotype

It is well established that TME polarizes macrophages toward the M2 phenotype, a phenotype with immunosuppressive, anti‐inflammatory, and pro‐angiogenic functions, and tumor‐associated macrophages (TAM) produce TGF‐β as one of the main immunosuppressive cytokines [159]. Lung‐resident tissue macrophages, through TGF‐β expression, generate Foxp3^+^ Tregs to promote tolerance to airway antigens, suggesting that blockade of TGF‐β is not optimal due to the critical functions in lung homeostasis [160]. During inflammation, TGF‐β acts as a chemoattractant for monocytes and increases adhesion molecules that enable monocyte attachment to the extracellular matrix [161, 162]. Subsequently, monocytes differentiate into macrophages and promote blood vessel leakiness, facilitating tumor cell extravasation [163]. TGF‐β1 in macrophages blocks the NF‐κB activation through myeloid differentiation factor 88 (MyD88), key adaptor protein required for NF‐κB nuclear translocation, ubiquitination and proteasomal degradation [164] and via SMAD6‐smurf‐mediated MyD88 degradation [165], thus inhibiting anti‐inflammatory responses. Furthermore, TGF‐β via SMAD7 suppresses inflammatory phenotype of macrophages by crosstalk with TNF signaling pathway, contributing to immune evasion by dampening the inflammatory functions of macrophages [166].

Recent studies indicate that TGFβ‐dependent antitumor responses of macrophages are an important and clinically relevant mechanism by which lung tumor growth is regulated. Standiford *et al*. reported that TAMs express significantly higher levels of interleukin receptor‐associated kinase (IRAK)‐M, a potent negative regulator of TLR signaling expressed in macrophages, compared with peritoneal macrophages in mice models of lung cancer. Also, IRAK‐M gene expression in adenocarcinoma tumors was associated with poor survival in lung cancer patients. Interestingly, the tumor cell‐induced term of IRAK‐M depended on activating the TGF‐β pathway and treating human PBMCs or mouse macrophage cell lines with TGFβ‐induced IRAK‐M expression [167]. Another study revealed that TAMs promote tumor metastasis via the TGFβ/SOX9 axis in NSCLC clinical samples. Abnormal SOX9 expression occurs in different types of cancers, and thereby, the TGFβ/SOX9 axis may be an effective therapeutic target for lung cancer [78]. An improved understanding of the role of TGF‐β in temporal dynamics of different TAM subsets and molecular mechanisms involved in the mediation of inflammatory functions of macrophages in lung cancer will be crucial for optimizing the targeting of TAMs for therapeutic benefit (Fig. 7C).

### Tumor microenvironment

Besides direct stimulation of tumor invasion and progression, TGF‐β can also indirectly promote the spread of cancer cells by regulating tumor microenvironment [9, 80]. TGF‐β promotes tumor stroma development, angiogenesis, and remodeling of the extracellular matrix [168, 169]. Poor prognosis in patients with surgically resected small peripheral adenocarcinomas was correlated with differences in stroma characteristics [170] that could be due to TGFβ activity. The *tumor stroma* comprises a basement membrane, cancer‐associated fibroblasts, an extracellular matrix, immune cells, and blood vessels [171]. In lung cancer, TGFβ‐induced release of some stimulators, such as forkhead box F1 from fibroblasts, was found to promote tumor progression and remodeling of ECM [172, 173]. Also, TGFβ‐induced release of angiogenic factors, such as vascular endothelial growth factor and connective tissue growth factor, was found to promote angiogenesis. In contrast, lymph angiogenesis was suppressed by TGF‐β [174]. It was found that TGF‐β produced by cancer cells induced the polarization of immune cells, such as macrophages, suppressor cells, and neutrophils, in the tumor microenvironment [175, 176].

## Potential TGF‐β‐associated therapeutics for lung cancer

**Fig. 8:**
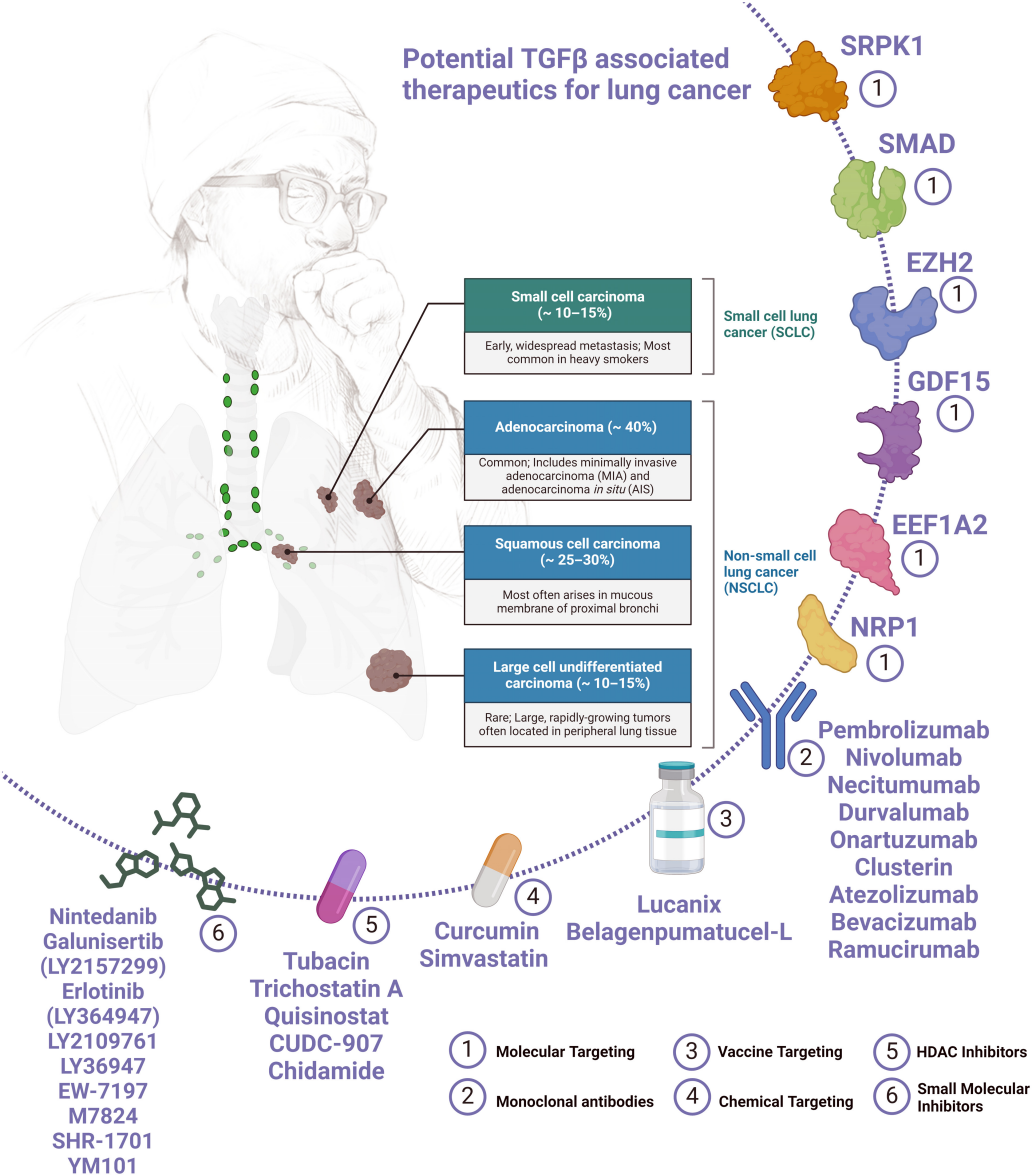
Potential TGFβ‐related lung cancer therapies. TGF‐β is a cytokine that controls several biological processes, especially at the pulmonary level; it has been shown that TGF‐β alterations are linked to lung cancer development. In addition, TGF‐β plays a significant role in driving EMT induction, promoting invasiveness and metastasis [9, 177]. Consequently, a TGF‐β signature may be used as a prognostic marker for lung cancer [90, 104]. TGF‐β signaling is pleiotropic, meaning that it affects many different cellular processes. This negatively affects drug resistance, tumor escape, immunosurveillance, and clinical response to treatment. The TGF‐β pathway has been pharmacologically targeted using TGFβ‐X inhibitors (X: SRPK1, SMAD, EZH2, GDF15, EEF1A2, NRP1), monoclonal antibodies, immune checkpoint inhibitors, vaccines, phytochemical drugs, HDAC inhibitors, and small molecule inhibitors. There are still major obstacles to overcome even though the TGF‐β pathway antagonists are fast emerging as extremely promising, safe, and effective anticancer medicines. The clinical development of TGF‐β pathway antagonists has been slowed by the difficulty of minimizing the unintended reduction of tumor‐suppressing activity and inflammatory consequences while maintaining the targeted limitation on tumor‐promoting activities (Created with BioRender.com) [177].

The crucial role of TGF‐β in lung‐related tumors, specifically NSCLC and lung fibrosis, has been validated for a long time. Despite understanding the major molecular components of the TGF‐β pathways in cancer, how targeting this pathway could assist with cancer therapy still needs to be elucidated. Various therapeutic targets could be applied to the TGF‐β signaling pathway with different mechanisms for tumor therapy purposes [90]. This section reviews the TGF‐β signaling pathway promising to target approaches in alleviating lung‐related morbidities, either malignant tumors or other benign lung morbidities (Fig. 8).

### Molecular targeting

SRPK1 (serine/arginine‐rich protein‐specific kinase 1), EZH2 (enhancer of zeste homolog 2), GDF15 (growth differentiation factor 15), EEF1A2 (eukaryotic translation elongation factor 1 alpha 2), and NRP1 (neuropilin 1) represent crucial targets in cancer therapy, particularly within the context of TGF‐β signaling pathways driving tumor progression. SRPK1, a protein kinase central to alternative splicing regulation, is implicated in cancer development and progression. Inhibition of SRPK1 holds promise in hindering tumor growth and metastasis [178, 179]. EZH2, a histone methyltransferase, silences genes by methylating histone H3 at lysine 27 (H3K27), and its overexpression is linked to various cancers. Targeting EZH2 with inhibitors offers potential for cancer therapy by reversing its epigenetic effects [180]. GDF15, belonging to the TGF‐β superfamily, impacts cancer progression by enhancing tumor growth, metastasis, and drug resistance through modulation of TGF‐β signaling pathways [181]. EEF1A2, involved in protein synthesis, is associated with increased tumor aggressiveness when overexpressed. Strategies targeting EEF1A2 function hold promise as a therapeutic approach in cancer treatment [182]. NRP1, a transmembrane protein, promotes angiogenesis and tumor growth by interacting with TGF‐β signaling pathways. Investigating NRP1 as a therapeutic target offers a potential avenue for cancer treatment [183]. Overall, understanding and targeting these molecules provide diverse opportunities for intervening in cancer progression, with the aim of improving outcomes for patients through inhibiting tumor growth, metastasis, and aggressiveness.

#### SRPK1 targeting

The SRPK1 pathway plays a critical role in initiating cancer by influencing cell survival mechanisms and promoting aggressive cancer traits [184, 185]. SRPK1 mediates TGFβ‐induced apoptosis by regulating JNK and AKT, echoing findings where Daxx, a protein linked with the Fas receptor and associated with JNK activation, has been directly tied to the TGF‐β apoptotic‐signaling pathway [186]. SRPK1 inhibition suppresses angiogenesis, metastasis, and the acquisition of a cancer stem cell phenotype, enhancing apoptosis in lung cancer [187, 188] and reducing the survival of cancer cells [189]. Research has shown that abnormal levels of SRPK1 are linked to worse outcomes in NSCLC by enhancing cell growth and resistance to chemotherapy. Targeting SRPK1 with specific antibodies can lower its levels and reduce tumor spread and invasion in lung cancer. Additionally, SRPK2, a related protein, helps cancer cells multiply and progress through the cell cycle by activating a key regulatory protein, E2F1 [94]. Overactive SRPK1 and SRPK2 are associated with more aggressive cancer and increased drug resistance [190]. Notably, SRPK1, when aberrantly expressed in either direction, induces constitutive Akt activation, a known oncogenic hallmark [191]. Overactive SRPK1 and SRPK2 are associated with more aggressive cancer and increased drug resistance [190]. Reducing SRPK1 expression can make cancer cells more responsive to chemotherapy and decrease their invasive capabilities, highlighting its potential as a target for new cancer therapies [192]. SRPK1 promotes a stem cell‐like phenotype in NSCLC through the Wnt/β‐catenin signaling pathway [193]. Chimeric antibody targeting SRPK‐1 inhibited mRNA expression levels of β‐catenin/T‐cell factor, matrix metalloproteinase‐9 (MMP‐9), collagen type I, and fibronectin in NSCLC‐derived vascular endothelial cells [194].

#### Targeting TGFβ/SMAD signaling pathway

Targeting the TGFβ/SMAD signaling pathway entails concentrating on particular proteins, known as inhibitory SMADs, specifically SMAD6 and SMAD7. These proteins directly control the activity of TGF‐β receptors by ubiquitinating and degrading the TGFβRI receptor, which generally facilitates the growth and spread of lung cancer [195, 196, 197]. Specifically, SMAD6 targets pathways outside the main TGF‐β signaling route by blocking a series of interactions (TRAF6‐TAK1‐p38 MAPK/JNK) that would otherwise lead to cancer progression, aided by the A20 enzyme that removes ubiquitin labels from proteins [42]. The intricate environment within lung tissues, influenced by elements such as pollutants or chronic inflammation, necessitates advanced delivery methods for treatments. For instance, research demonstrates that nanoparticles loaded with the drug pirfenidone can reverse EMT by modulating the TGF‐β1 and SMAD signaling pathways, establishing these small carriers as crucial for effective treatment in these conditions [198]. The canonical TGFβ/SMAD signaling pathway is also involved in programmed death ligand‐1 (PD‐L1)‐induced primary resistance to epidermal growth factor receptor‐tyrosine kinase inhibitors (EGFR‐TKIs) in EGFR‐mutant NSCLC [199]. Furthermore, the influence of TGF‐β, particularly regarding its EMT mechanisms, signifies its pivotal role in lung cancer progression, presenting opportunities for therapeutic interventions [90].

#### SMASR

A TGFβ/SMAD signaling pathway is essential in EMT during lung cancer progression [200]. A novel long non‐coding RNA adjacent to SMAD3, named SMAD3‐associated long non‐coding RNA (SMASR), is downregulated by TGF‐β through SMAD2/3 in lung cancer cells. Importantly, SMASR inhibits the TGFβRI by interacting with Smad2/3, leading to the inactivation of the TGFβ/SMAD signaling pathway and forming a negative feedback loop, which plays a critical role in regulating EMT in lung cancer. This evidence suggests that SMASR could be applicable as a novel EMT marker and a promising lung cancer therapeutic [201].

#### EZH2 inhibitors

TGFβRII is expressed at low levels in most small‐cell lung cancer (SCLC) cells compared to normal lung epithelial cells and lung tissues. Enhancer of zeste homolog 2 (EZH2) expression in SCLC cells is associated with the epigenetic silencing of TGFβRII expression, TGFβ‐SMAD‐ASCL1 (achaete‐scute family bHLH transcription factor 1) pathway suppression, and contributing to SCLC progression. Thus, EZH2 polycomb repressive complex 2 subunit inhibitors are proposed to target SCLC lung cancer [202]. Also, overexpression of EZH2 is linked with a worse prognosis in lung cancer, particularly showing higher levels in late‐stage NSCLC than in early stages [203]. Further studies reveal the interaction between YAP/TAZ and cancer‐promoting microRNAs (miR‐25, miR‐93, and miR‐106b) in NSCLC, identifying TGFβRII as a direct target of this miRNA group, which suggests a potential tumor‐suppressing role for TGFβRII. The inhibition of TGFβRII by YAP/TAZ, through both transcriptional and post‐transcriptional methods, along with research indicating that an increase in TGFβRII expression could improve TGF‐β sensitivity and reduce lung tumor growth in both NSCLC and small‐cell lung cancer (SCLC) cell lines, underscores the complexity of these pathways [204].

#### GDF15

Growth differentiation factor 15 (GDF15), a member of the TGF‐β superfamily of cytokines, is a therapeutic protein for NSCLC treatment. Further to its role as a growth differentiation factor, GDF15 also functions as macrophage inhibitory factor 1, influencing cell metabolism, inflammation, and immune escape [205, 206]. Research has shown that GDF15 decreases cell proliferation, migration, and invasion in the adenocarcinoma human alveolar basal epithelial cell line (A549) and significantly reduces lung and bone metastases in animal models [207]. GDF‐15‐induced apoptosis in human non‐small‐cell lung carcinoma cell line (H460) is mediated through CD437 (a selective retinoic acid receptor agonist) and ST1926 (a synthetic retinoid with antitumor properties) [208]. Notably, GDF15 has been shown to exhibit dual roles during carcinogenesis, inhibiting tumor growth in the initial stages while promoting proliferation in later stages. In A549 cells, the apoptosis‐inducing effect of GDF15, as well as its influence on MAPK activation, is contingent upon TGFβRII expression, with the absence or silencing of TGFβRII mitigating GDF15's pro‐apoptotic activities [209, 210]. GDF15 downregulation in NSCLC is associated with a poor prognosis [208], and its serum levels could be applied as a predicting factor for lung cancer diagnosis and the efficacy of chemotherapy [211]. Furthermore, GDF15 is an important downstream component of the C2 domain‐containing phosphoprotein (CDP138), which impacts the TGFβ/SMAD signaling pathway, essential for controlling tumor resistance to radiation and its potential to metastasize [212].

#### EEF1A2 silencing

Suppressing the oncogene eukaryotic protein translation elongation factor 1α2 (EEF1A2) is essential for facilitating epithelial–mesenchymal transition (EMT). This process is mediated by elevated levels of key TGF‐β signaling components—TGFβRI, TGFβRII, SMAD3, and phosphorylated SMAD3—which subsequently translocate to the nucleus to exert their influence. EEF1A2 expression is associated with lung adenocarcinoma metastasis, poor prognosis, tumor grade, disease stage, and short survival of suffering patients [213]. Interestingly, the suppression of the EEF1A2 oncogene diminishes the invasive, migratory, and proliferative capabilities of A549 lung cancer cells. This decrease correlates with reduced levels of TGFβRI and TGFβRII receptors and alterations in SMAD3 and its phosphorylated variant, pSMAD3, further illustrating the contribution of EEF1A2 to increasing tumor aggressiveness [214].

#### Neuropilin‐1

Neuropilin‐1 (NRP1) protein promotes the radiation‐induced EMT through TGFβ/SMAD signaling pathway in lung adenocarcinoma cells [215]. Additionally, NRP1 significantly impacts how cells respond to TGF‐β1 by interacting with various SMAD proteins. This interaction influences the development of myofibroblast phenotypes, a key process in fibrosis and cancer progression [216]. Also, NRP1 contributes to TGFβ1‐induced EMT and metastasis in NSCLC by binding directly to the TGFβRII receptor, further illustrating its role in aggressive cancer behaviors [217]. According to these findings, NRP1 could be considered as a clinical detection marker and a target for molecular‐targeted therapy of NSCLC.

### Monoclonal antibodies

Several approved monoclonal antibodies have shown promises in the treatment of NSCLC. Agents like pembrolizumab and nivolumab work by targeting PD‐1 molecule, leading to the induction of programmed tumor cell death. Necitumumab, durvalumab, and onartuzumab are other notable monoclonal antibodies used in NSCLC management.

A key molecular target in the context of NSCLC is Clusterin, which acts as an important extracellular promoter of EMT. The Clusterin gene gets significantly upregulated by TGF‐β, leading to an increased secretion of Clusterin.

Apart from the antibodies mentioned above, atezolizumab is another monoclonal antibody that binds to PD‐L1, inhibiting its interactions with PD‐1 and B7‐1 receptors. This blockade enhances the activation and proliferation of T cells that target cancer [218]. Similarly, bevacizumab is an antibody that targets vascular endothelial growth factor (VEGF) and has been shown to improve survival rates when combined with traditional chemotherapy for NSCLC [219]. Ramucirumab, targeting VEGF receptor 2, has also been approved for use with docetaxel in patients with metastatic NSCLC after disease progression on platinum‐based chemotherapy [220]. Moreover, amivantamab, a fully human bispecific antibody that inhibits ligand binding through EGFR and MET receptor, demonstrated antitumor potential against diverse EGFR‐ and MET‐driven NSCLC. Amivantamab has been approved for metastatic NSCLC (harboring EGFR exon20ins mutations). Amivantamab is currently investigated in trials for combinatorial therapies in NSCLC patients with further chemotherapeutics [221]. These therapies exemplify the broadening horizon of immunotherapeutic strategies aiming diverse targets for more efficient NSCLC management.

#### Dual targeting of PD‐1/PD‐L1 and TGFβ/TGFβR pathways

Antitumor immune responses induced by immune checkpoint inhibitors, including anti‐PD‐1 or anti‐PD‐L1, are considered therapeutic strategies in advanced NSCLC. Programmed cell death‐protein 1/programmed death‐ligand 1 (PD‐1/PD‐L1) inhibitors, including bintrafusp alfa (M7824), SHR‐1701, and YM101, were developed to block PD‐1/PD‐L1 and TGFβ/TGFβR receptors simultaneously. Studies have shown that lung cancer patients with poor lymphocyte recovery may resist anti‐PD‐1/PD‐L1 antibodies but might be sensitive to SHR‐1701 [222]. Targeting TGFβ trap to PD‐L1+ endothelium and the M2/lipofibroblast‐like cell compartment by bintrafusp alfa has improved radiotherapy‐induced lung fibrosis [223]. Favorable antitumor activity to immune checkpoint inhibitors is correlated with high PD‐L1 expression, increased tumor‐infiltrating lymphocytes, and decreased Treg cells or tumor‐associated macrophages. It has been shown that higher levels of TGF‐β can also lead to a positive prognosis for anti‐PD‐1 immunotherapy in advanced NSCLC patients [141].

### Vaccines targeting

Cancer stem cells (CSCs) are the most common causes of lung cancer relapse and resistance to chemotherapy. Thus, vaccines targeting CSC are versatile therapeutic tools in lung cancer. In this regard, the dendritic cell vaccine loaded with tumor cell lysate showed amelioration of cancer stemness by modulating lung tumor immune archetypes. These processes were indicated by an increased percentage of CD8^+^, CD83^+^, and IL‐12, as well as downregulation of TGF‐β, PD‐L1, CTLA‐4, and Foxp3 gene expression on the percentage of CD166^+^ CSC in the lung of mice exposed to Benzo(a) Pyrene [224]. Polyinosinic–polycytidylic acid (polyI:C), the synthetic agonist for toll‐like receptor (TLR) 3, suppressed the motility of A549 cells undergoing EMT through targeting the TGFβ1‐induced phosphatidylinositol 3‐kinase/Akt pathway [225]. The whole‐cell vaccine developed by fusing HUVEC and A549 cells showed inhibition of tumor angiogenesis by diminishing VEGF and TGFβ angiogenic mediators and Tregs. Further, this cell‐based vaccine robustly inhibited lung metastasis in C57BL/6 murine lung carcinoma model (LL (2)) [226].

In a gene therapy vaccine approach, IL‐7 potential was exploited to downregulate tumoral TGF‐β and stimulate CTL responses. The multicomponent vaccine comprising of *ex vivo* transduced autologous tumor cells with a retroviral vector (containing both IL‐7 and HyHSVtk (herpes simplex thymidine kinase‐transduced genes)) as well as *ex vivo* activated DC effectively eradicated lung tumor tissue [227]. In a phase I trial clinical study, expression plasmids of both GM‐CSF and TGF‐β2 antisense nucleotide sequences were incorporated into an autologous whole‐cell vaccine, considering that the combined expression could break tolerance and stimulate immune responses [228]. Belagenpumatucel‐L, a nonviral TGF‐β2 antisense gene‐modified allogeneic tumor vaccine designed to enhance tumor antigen recognition by virtue of TGF‐β2 inhibition, showed safety in phase II advanced NSCLC clinical trial and further showed enhanced survival rate [229, 230]. In the phase III clinical trial, Belagenpumatucel‐L was well tolerated, with no serious safety concerns within the 12‐week treatment course. Data analysis indicated that the prior radiation therapy in some patients was a positive prognostic factor in the patients treated with this therapeutic [231]. Clinical studies on Lucanix™, a TGF‐β antisense gene and nonviral gene‐based allogeneic tumor cell vaccine, are in progress for NSCLC treatment. In the preclinical stages, this vaccine showed enhanced tumor antigen recognition as the consequence of TGF‐β2 inhibition, and it showed both safety and well tolerability in the phase II trial [232].

### Chemicals targeting

#### Curcumin

Curcumin is a leading phytochemical that mitigates most inflammatory immune‐related disorders, mainly cancers, by regulating various signaling pathways [233, 234]. In lung cancer, curcumin exerts its antitumoral effects via modulating different agents, including TGF‐β. Curcumin could also regulate the TGF‐β signaling pathway in scleroderma fibroblasts, neonatal lung fibroblasts, and renal cells. Curcumin is a potential anticancer agent for both TGF‐β‐sensitive and TGF‐β‐resistant NSCLC tumors [235]. Of note, reports have also shown that curcumin could exert its antimetastatic effect by modulating the TGF‐β1‐induced EMT process in lung cancer cells. Curcumin can mitigate EMT by regulating the expression of crucial mesenchymal markers (e.g., MMP2, vimentin, and N‐cadherin) [236].

#### Statins

Simvastatin, a chemical immunomodulatory agent, is known to enhance TGF‐β production and Treg proliferation as observed in the 3LL and NCI‐H292 cell lines; however, it does not exert this effect in A549 cells. Moreover, simvastatin did not affect tumor growth in a 3LL cell‐inoculated mouse tumor model, indicating limitations in its potential for tumor treatment [237]. The role of atorvastatin in cancer treatment is associated with its modulation of the EMT rather than direct inhibition of the TGF‐β/Smad2/3 signaling pathway. It has been demonstrated that atorvastatin can mitigate EMT in NSCLC, which may be related to its ability to attenuate the upregulation of sphingosine kinase 1 (SphK1) that is induced by TGF‐β1. This action suggests an indirect mechanism by which atorvastatin, along with simvastatin—both HMG‐CoA reductase inhibitors—may exert broad effects that potentially interfere with complex biological responses such as EMT [238].

### Histone deacetylase (HDAC) inhibitors

Dysregulation of Notch signaling is a common feature of NSCLC and is correlated with poor prognosis of lung cancer. HDAC6 is a key regulator of TGFβ‐induced EMT in NSCLC. Thus, HDAC inhibitors are attractive cancer therapeutics, inducing tumor cell apoptosis, differentiation, senescence, growth arrest, and angiogenesis inhibition [239, 240]. Tubacin, an HDAC6 inhibitor small molecule, attenuated TGFβ1‐induced Notch‐1 signaling [241]. Trichostatin A and quisinostat are also HDAC inhibitors that downregulate the tight junction proteins of human lung adenocarcinoma cells (A549) and normal lung epithelial cells through TGFβ‐induced cellular metabolism [242]. Inhibition of lung cancer adenocarcinoma cells by trichostatin A was potentiated by peroxisome proliferator‐activated receptor‐γ ligands, resulting in decreased expression of cyclin D1 [243].

CUDC‐907 functions as a dual inhibitor of HDAC and PI3K/AKT pathways. It has been found that CUDC‐907 inhibits myofibroblasts/CAF cell migration and proliferation and significantly increases apoptosis in a dose‐dependent manner, leading to cell cycle arrest. CUDC‐907 also inhibits expression of myofibroblast markers, reduces phosphorylation levels of AKT, mTOR, and SMAD2/3, and promotes histone acetylation. Therefore, CUDC‐907 could be considered a treatment strategy for TGFβ1‐induced lung and tumor fibrosis. TGF‐β1 signaling pathway is a critical driver of collagen accumulation in pulmonary fibrotic morbidities. It is also considered a regulator of cancer‐associated fibroblast (CAF) activation in lung cancer by simultaneously targeting PI3K (phosphoinositide 3‐kinases) and HDAC pathways in lung myofibroblasts. Thus, CUDC‐907, a dual inhibitor of both PI3K/Akt/mTOR and HDAC, could be considered an efficient strategy to treat lung fibrosis by suppressing collagen production, ECM deposition as well as inhibiting the invasion of CAF [244]. Chidamide, an HDAC1 inhibitor, prohibits TGFβ‐induced SMAD2 phosphorylation and attenuation of TGFβ‐induced loss of E‐cadherin expression, leading to the inhibition of migration in lung cancer cells [245]. HDAC inhibitors can restore TGFβ‐induced tumor suppressor function in lung cancer cell lines that lack TGFβRII expression [246].

### Small molecular inhibitors

Nintedanib is a small molecular inhibitor approved for treating idiopathic pulmonary fibrosis (IPF). In some countries, nintedanib is also co‐administered with docetaxel for patients with advanced NSCLC after first‐line chemotherapy [247]. This therapeutic offers antifibrotic action against the TGF‐β pathway in NSCLC tumor‐associated fibroblasts [248]. This drug can also inhibit the TGFβ1‐induced expression of pro‐fibrotic activation markers in adenocarcinoma tumor‐associated fibroblast parenchyma [249]. Nintedanib also inhibits EMT by mediating EMT‐related gene expression and the TGFβ/SMAD pathway in A549 cells [250]. Nintedanib monotherapy could be a therapeutic option for NSCLC patients with idiopathic pulmonary fibrosis and chemotherapy intolerance [251]. When this small molecular inhibitor was linked to αvβ6 receptor, it reduced TGFβ‐induced EMT in human NSCLC cells, highlighting the role of αvβ6 receptors as an efficacious targeting approach against NSCLC [252].

Galunisertib (LY2157299), an orally administered small molecule that inhibits TGFβRI kinase and TGF‐β, was extensively evaluated on various NSCLC or SCLC tumor models (either patient‐derived xenografts or cell line‐derived xenografts) [253]. However, galunisertib development by Eli Lilly was discontinued in January 2020 due to other new R&D priorities [254].

Erlotinib (LY364947) is a pyrazole‐based small molecular inhibitor capable of inhibiting the serine–threonine kinase activity of TGFβRI. In various studies, erlotinib monotherapy did not show any antitumor effect, whereas its combined treatment with anti‐PD‐L1 mAb significantly delayed tumor progression [255]. TGF‐β signaling pathway blockade combined with continuous epidermal growth factor receptor tyrosine kinase inhibitor (EGFR TKI) treatment could be beneficial in preventing metastasis in patients with NSCLC resistant to erlotinib [256]. As another small molecule inhibitor, crizotinib, a tyrosine kinase inhibitor harboring EML4‐ALK fusion, has been found to attenuate metastasis by inhibiting TGF‐β signaling in NSCLC cells without affecting cell growth [257]. Also, it has shown to exert its antitumoral activity via blocking TGFβ‐induced SMAD activation, suppressing TGFβ‐ and HGF‐induced cell invasion and migration in NSCLC cells, and directly inhibiting TGFβRI kinase activity in a competitive inhibitory manner [257].

A TGF‐β signaling pathway is vital in radiotherapy‐induced acute pneumonitis and pulmonary fibrosis. Oral administration of a novel small‐molecule TGFβRI serine/threonine kinase inhibitor (LY2109761) to C57BL/6 mice (a single dose for 4 weeks before, during or after radiotherapy) markedly reduced pulmonary fibrosis/inflammation and led to prolonged survival. It was found that LY2109761 can reduce phosphorylation of SMAD2 and SMAD1. It also suppresses the expression of genes involved in canonical and noncanonical TGF‐β signaling and radiation‐induced inflammatory cytokines, such as IL‐6, IL‐7R, and IL‐8. These data revealed that LY2109761 achieves its antifibrotic effects through radiation‐induced proangiogenic, proinflammatory, and profibrotic signal suppression [258]. After radiation therapy, pretreatment of several lung‐related tumor cells with LY364947 attenuated DNA damage with decreased p53 phosphorylation and increased radiosensitivity [259]. In the context of therapeutic alternatives for lung conditions, the EMERGING‐CTONG 1103 trial demonstrated that neoadjuvant erlotinib can improve the outcomes in Stage IIIA‐N2 NSCLC patients with EGFR mutations, presenting better tolerability over gemcitabine‐cisplatin chemotherapy with postoperative therapy showing no severe adverse events [260].

Another novel synthesized small molecule inhibitor of the TGFβRI, named EW‐7197, has showed strong potential as an antifibrosis therapeutic by inhibiting TGFβ/SMAD2/3 and ROS signaling [261]. Transactivation of the TGF‐β pathway can also initiate the fibroblast activation by TGFβRI [262]. Inhibiting the kinase activity of budding uninhibited by benzimidazoles‐1 (BUB1), as a critical mediator of TGF‐β signaling, has been found to suppress TGF‐β signaling in various normal and cancer cells as well as mice bearing lung carcinoma xenografts and to reduce the amounts of phosphorylated SMAD2 in lung carcinoma tumor tissue [263]. Research utilizing multiple databases and *in vitro* experiments revealed that BUB1 overexpression is an adverse prognostic factor in lung adenocarcinoma. It promotes tumor cell proliferation, migration, and invasion, thus signifying its potential as a therapeutic target [264].

## TGF‐β pathway‐induced chemoresistance

Chemoresistance is a major cause of cancer mortality partly due to the limited accessibility to immune therapeutics or targeted therapies [265]. Upregulation of tumor cells PD‐L1 is considered a novel mechanism of TGFβ1‐induced immunosuppression in NSCLC. Treatment of NSCLC with bintrafusp alfa (M7824) targeting PD‐L1 and TGF‐β simultaneously has shown to block tumor mesenchymalization and PD‐L1‐dependent immunosuppression and dominated chemoresistance [266]. Bintrafusp alfa has also shown some antitumor activity in NSCLC patients either pretreated with anti‐PD‐(L)1 who displayed acquired resistance to prior therapy or who had primary refractory disease [267]. In another instance, elevated levels of miR‐128‐3p have been identified as significant contributors to both chemoresistance and metastasis in NSCLC, primarily by downregulating inhibitors of the Wnt/β‐catenin and TGF‐β pathways [268]. Also, myeloid leukemia 1 (MCL‐1) has been identified for chemoresistance domination in A549 cells associated with TGFβ‐induced EMT. Downregulation of MCL‐1 by siRNA or inhibition of MCL‐1 diminishes the EMT‐associated chemoresistance in A549 cells [269].

Radiotherapy of lung carcinoma has shown hyperactivation of TGFβ signaling, which contributes to EMT‐associated changes (i.e., metastasis, cancer stem cell formation, and chemoresistance) [270]. It has been validated that high levels of miR‐128‐3p are responsible for the concurrent development of chemoresistance and metastasis in NSCLC [271]. By targeting and reversing miR‐128‐3p, it could be possible to potently change metastasis and chemoresistance in malignant NSCLC cells [268]. Further studies revealed that EMT‐induced chemoresistance could be successfully reverted by EMT‐reverting agents, including metformin, PP242 (mTOR inhibitor), and DMSO [272].

Epithelial splicing regulatory protein 1 (ESRP1), an RNA‐binding protein that regulates mRNA splicing, is partially responsible for the chemoresistance of various cancers, including NSCLC [273]. ESRP1 is significantly downregulated in SCLC chemo‐resistant cells. ESRP1 overexpression has been found to increase SCLC chemosensitivity and induce cell apoptosis and cell cycle arrest, while the knockdown of ESRP1 acts controversial. ESRP1 inhibits the TGFβ/SMAD signaling pathway by regulating alternative splicing of CARM1, thereby reversing the chemoresistance of SCLC. The splicing factor ESRP1 may be a new therapeutic molecule and resistance marker for targeting SCLC [274]. Circular RNA ESRP1 plays a crucial role in SCLC‐chemosensitivity through miR‐93‐5p in order to inhibit the TGF‐β pathway. These findings show that cESRP1 may serve as a prognostic biomarker and a potential therapeutic target in SCLC. Circular ESRP1 enhanced chemotherapeutics (doxorubicin, cisplatin, and etoposide) sensitivity by repressing miR‐93‐5p in SCLC [275].

## TGFβ‐induced immune modulation in lung cancer

The role of TGF‐β in the pathogenesis of lung cancer cannot be understated without investigating its profound effect on immune modulation which stands out as a cornerstone in the biology of the tumor. Within the tumor microenvironment, TGF‐β orchestrates a myriad of immunosuppressive mechanisms that pose significant hurdles for therapeutic interventions [276]. TGF‐β impairs T‐cell activation and differentiation, hinders the cytotoxic activities of natural killer cells, and compromises the antigen‐presenting capabilities of dendritic cells [277, 278, 279]. High levels of TGF‐β in the tumor microenvironment are often associated with a shift from a Th1 pro‐inflammatory milieu, crucial for effective antitumor immunity, to a Th2 immunosuppressive one, facilitating tumor evasion from immune surveillance [276]. TGFβ can stimulate the recruitment and proliferation of regulatory T cells (Tregs) and myeloid‐derived suppressor cells (MDSCs), both of which further dampen antitumor immune responses [280, 281]. Emerging evidence also underscores the potential implications of TGFβ‐mediated immune modulation on the efficacy of immunotherapeutic strategies [282, 283]. Some studies indicate that tumors with heightened TGF‐β signaling may be less responsive to checkpoint inhibitors, shedding light on potential combination therapeutic approaches that target both TGF‐β pathways and immune checkpoints [159, 284].

Given the revolutionary advancements in cancer immunotherapy, unraveling the nuances of TGF‐β's influence on the immune landscape is imperative. As we transition into the discussion on TGFβ‐based immunotherapies in the following sections, the intricate dance between TGF‐β and immune modulation will take center stage in dictating therapeutic outcomes.

## TGFβ‐based immunotherapy

Many studies have been conducted in the last decade to develop immunotherapeutic targeting TGF‐β for the treatment of varying types of cancers. Although many drugs have been developed to target TGF‐β in lung cancer, most have been discontinued. Currently, a limited number of agents that target TGF‐β are in clinical development (Tables 2 and 3).

**Table 2:** Ongoing and completed clinical trials assessing the efficacy and safety of transforming growth factor‐β (TGF‐β) inhibitors in lung cancer.

Study NCT number	Cancer type (number of patients)	Phase	Test drug	Targets	Efficacy
NCT01058785	NSCLC (*n* = 75)	Two	Belagenpumatucel‐L	Cancer vaccine	Stage III/IV: ORR 15%
NCT00676507	Inoperable or metastatic NSCLC after frontline platinum therapy (*n* = 532)	Three	Belagenpumatucel‐L versus placebo	Cancer vaccine (Lucanix™)	no significant difference in survival rate between the vaccine and placebo
NCT03732274	NSCLC (*n* = 26)	One/Two	Vactosertib combined with durvalumab	TβRI (ALK5)	Interim results: manageable safety profile and encouraging antitumor activity
NCT03631706	Untreated advanced NSCLC (*n* = up to 584)	Three	Bintrafusp alfa versus pembrolizumab	TbRII and PD‐L1 and PD‐1	Ongoing (https://www.clinicaltrials.gov/ct2/show/NCT03631706)
NCT03840915	Metastatic NSCLC, Stage IV	One/Two	Platinum‐based regimen + bintrafusp alfa	Chemotherapy TbRII and PD‐L1	Ongoing (https://www.clinicaltrials.gov/ct2/show/study/NCT03840915)
NCT02581787	Stage Ia/Ib NSCLC	One/Two	Fresolimumab + SBRT	TGFβ1, TGFβ2, and TGFβ3 RT	Ongoing (https://www.clinicaltrials.gov/ct2/show/NCT02581787)
NCT03631706	Untreated advanced NSCLC	Three	Bintrafusp alfa versus pembrolizumab	TβRII and PD‐L1 + PD‐1	Ongoing (https://www.clinicaltrials.gov/ct2/show/NCT03631706)

**Table 3:** Potential therapies for lung cancer associated with TGF‐β.

Medication name	Mechanism of action	Targeted pathway	References
Pembrolizumab, Nivolumab, Necitumumab, Durvalumab and Onartuzumab	Targets PD‐1 molecule, induces programmed tumor cell death	PD‐1 pathway	[285]
Atezolizumab	Binds to PD‐L1, inhibits PD‐L1 interactions with PD‐1 and B7‐1	PD‐L1 pathway	[218]
Bevacizumab	Targets vascular endothelial growth factor (VEGF)	VEGF pathway	[219]
Ramucirumab	Targets VEGF receptor 2	VEGF receptor 2 pathway	[220]
Amivantamab	Bispecific antibody inhibiting EGFR and MET receptors	EGFR and MET pathways	[221]
Bintrafusp alfa (M7824), SHR‐1701 and YM101	Blocks PD‐1/PD‐L1 and TGFβ/TGFβR receptors simultaneously	PD‐1/PD‐L1 and TGFβ/TGFβR pathways	[222]
Polyinosinic‐polycytidylic acid (polyI:C)	Synthetic agonist for toll‐like receptor 3	TGFβ1‐induced phosphatidylinositol 3‐kinase/Akt pathway	[225]
Belagenpumatucel‐L	Nonviral TGF‐β2 antisense gene‐modified allogeneic tumor vaccine, virtue of TGF‐β2 inhibition	TGF‐β2 pathway	[229, 230]
Lucanix™	Enhanced tumor antigen recognition	TGF‐β2 inhibition	[232]
Curcumin	Modulation of various signaling pathways, including TGF‐β; inhibition of EMT process in lung cancer cells	TGF‐β1 signaling pathway, EMT	[235, 236]
Simvastatin	Enhancement of TGF‐β production and Tregs proliferation	TGF‐β1 production, Tregs proliferation	[237]
Atorvastatin	Lowering of TGF‐β1 signaling pathway stimulation; partial inhibition of TGF‐β1‐induced EMT process in NSCLC by attenuating SphK1	TGF‐β1 signaling pathway, EMT	[238]
Tubacin	Inhibition of TGFβ1‐induced Notch‐1 signaling	Notch‐1 signaling	[241]
Trichostatin A	Downregulation of tight junction proteins through TGFβ‐induced cellular metabolism, decreased expression of cyclin D1 by peroxisome proliferator‐activated receptor‐ γ ligands	Tight junction proteins, TGFβ‐induced cellular metabolism, peroxisome proliferator‐activated receptor‐ γ ligands	[242, 243]
Quisinostat	Downregulation of tight junction proteins through TGFβ‐induced cellular metabolism	Tight junction proteins, TGFβ‐induced cellular metabolism	[242]
CUDC‐907	Dual inhibition of HDAC and PI3K/AKT pathways; inhibition of myofibroblasts/CAF cell migration, proliferation, and apoptosis	HDAC, PI3K/AKT pathways, myofibroblasts/CAF	[244]
Chidamide	Inhibition of TGFβ‐induced SMAD2 phosphorylation and E‐cadherin expression loss; inhibition of migration in lung cancer cells	TGFβ‐induced SMAD2 phosphorylation, migration	[245]
Suberoylanilide hydroxamic acid (SAHA)	HDAC inhibitor	Cancer cell senescence induction and secretion of senescence‐associated secretion phenotype (SASP)	[286]
SAHA and EZH2 inhibitors	Increase in the nuclear pore density, suppress CCF generation and preventing SASP secretion	Induced cellular senescence	[286]
Nintedanib	Inhibition of TGF‐β pathway in NSCLC tumor‐associated fibroblasts; inhibition of EMT and pro‐fibrotic activation markers	TGF‐β pathway, EMT, pro‐fibrotic activation markers, TGFβ/SMAD pathway in A549 cells	[249, 250]
Galunisertib	Inhibition of TGFβRI kinase and TGF‐β; evaluated in various NSCLC or SCLC tumor models	TGFβRI kinase, TGF‐β	[253]
Erlotinib	Inhibition of serine–threonine kinase activity of TGFβRI; combination therapy with anti‐PD‐L1 mAb for delaying tumor progression	TGFβRI serine–threonine kinase activity	[255, 287]
Crizotinib	Inhibition of TGF‐β signaling, TGFβ‐induced SMAD activation, cell invasion, migration; direct inhibition of TGFβRI kinase activity	TGF‐β signaling, TGFβRI kinase activity	[261]
LY2109761	Inhibition of SMAD2 and SMAD1 phosphorylation; suppression of canonical and noncanonical TGF‐β signaling; reduction of inflammatory cytokines	SMAD2/SMAD1 phosphorylation, inflammatory cytokines	[258]
EW‐7197	Inhibition of TGFβ/SMAD2/3 and ROS signaling; antimetastatic activity; inhibition of fibroblast activation; enhancement of cytotoxic T lymphocyte activity	TGFβ/SMAD2/3 signaling, ROS signaling	[261, 262, 288]
Benzimidazoles‐1 (BUB1)	Suppression of TGF‐β signaling; reduction of phosphorylated SMAD2; inhibition of tumor cell proliferation, migration, and invasion	TGF‐β signaling, phosphorylated SMAD2	[263, 264]
Bintrafusp alfa (M7824)	Block tumor mesenchymalization and PD‐L1‐dependent immunosuppression and dominated chemoresistance	PD‐L1 and TGF‐β pathways	[266]

## Conclusion

The multifaceted nature of TGF‐β is deeply embedded in the realm of lung physiology and pathology, extending from its vital roles in lung development and homeostasis to the complexity of lung cancer dynamics [9, 75, 76]. As a result, TGFβ significantly influences carcinogenesis and lung cancer progression. In the early phases of malignancy, TGF‐β acts as a tumor suppressor, counteracting tumor growth, moderating cell proliferation, triggering apoptosis, and preserving harmony within the tumor microenvironment [90]. However, mutations that incapacitate its tumor‐suppressive mechanisms can inadvertently push the system toward tumorigenesis and expedite cancer progression. The protective mechanisms of TGF‐β are further undermined when tumor cells undergo epigenetic modifications, allowing them to evade the inhibitory actions of TGF‐β [289, 290]. This landscape presents a paradox since increased TGF‐β activity, rather than acting as a deterrent, accelerates tumor growth and paves the path for metastatic endeavors. Herein, the process of EMT, bolstered by TGF‐β, emerges as a critical facilitator of this cancerous advancement [89]. Beyond these cellular alterations, TGF‐β profoundly changes the tumor microenvironment and the complex network of immune interactions [91]. Through its sophisticated modulation of the immune milieu, TGF‐β offers a refuge for tumor cells, allowing them to bypass immune defenses while tailoring the microenvironment to favor tumor progression. This dual role of TGF‐β, as a protector and a promoter of tumor progression particularly its adeptness in altering immune responses and tumor microenvironment, anchors its pivotal role in lung cancer biology from initiation to metastatic spreading. Unraveling these layers of complexity is essential. As research continues to penetrate the intricacies of lung cancer biology, the promise of therapeutic interventions targeting the TGF‐β pathway becomes increasingly evident, offering novel opportunities for more precise and impactful treatments in the future. This hopeful perspective must be balanced with a realistic evaluation of the forthcoming obstacles. The complex role of TGF‐β in cancer pathophysiology presents promising opportunities for new treatments, yet caution is essential. The dual function of TGF‐β in both suppressing and promoting tumors complicates the development of effective therapies. Historically, many molecules have shown potential in preclinical studies but failed to yield successful treatments in humans. This inconsistency underscores the limitations of using mouse models to replicate human disease processes and the unpredictable nature of human biological responses. Therefore, while this review highlights the potential of targeting TGF‐β in lung cancer treatments, it also emphasizes the need for thorough and meticulous research that connects lab discoveries to clinical success. Future research in this area must carefully navigate the complexities of TGF‐β signaling and its overall effects to avoid the setbacks experienced by previous efforts and to genuinely enhance the pursuit of effective cancer treatments.

## Conflict of interest

The authors declare no conflict of interest.

## Author contributions

SA, ABB, NR, and MS contributed to conceptualization, writing—original draft preparation, writing—review and editing, and figure preparation. FA contributed to revision. MC and SP contributed to writing—review and editing. SHA, NT, and SG contributed to conceptualization, writing—review and editing, and supervision. All authors read and approved the final manuscript.
